# FGFR3 Deficiency Causes Multiple Chondroma-like Lesions by Upregulating Hedgehog Signaling

**DOI:** 10.1371/journal.pgen.1005214

**Published:** 2015-06-19

**Authors:** Siru Zhou, Yangli Xie, Junzhou Tang, Junlan Huang, Qizhao Huang, Wei Xu, Zuqiang Wang, Fengtao Luo, Quan Wang, Hangang Chen, Xiaolan Du, Yue Shen, Di Chen, Lin Chen

**Affiliations:** 1 Center of Bone Metabolism and Repair, Department of Rehabilitation Medicine, State Key Laboratory of Trauma, Burns and Combined Injury, Trauma Center, Institute of Surgery Research, Daping Hospital, Third Military Medical University, Chongqing, China; 2 Department of Biochemistry, Rush University Medical Center, Chicago, Illinois, United States of America; University of Oxford, UNITED KINGDOM

## Abstract

Most cartilaginous tumors are formed during skeletal development in locations adjacent to growth plates, suggesting that they arise from disordered endochondral bone growth. Fibroblast growth factor receptor (FGFR)3 signaling plays essential roles in this process; however, the role of FGFR3 in cartilaginous tumorigenesis is not known. In this study, we found that postnatal chondrocyte-specific *Fgfr3* deletion induced multiple chondroma-like lesions, including enchondromas and osteochondromas, adjacent to disordered growth plates. The lesions showed decreased extracellular signal-regulated kinase (ERK) activity and increased Indian hedgehog (IHH) expression. The same was observed in *Fgfr3*-deficient primary chondrocytes, in which treatment with a mitogen-activated protein kinase (MEK) inhibitor increased *Ihh* expression. Importantly, treatment with an inhibitor of IHH signaling reduced the occurrence of chondroma-like lesions in *Fgfr3*-deficient mice. This is the first study reporting that the loss of *Fgfr3* function leads to the formation of chondroma-like lesions via downregulation of MEK/ERK signaling and upregulation of IHH, suggesting that FGFR3 has a tumor suppressor-like function in chondrogenesis.

## Author Summary

Benign cartilaginous tumors, especially osteochondromas and enchondromas, are the most common primary bone tumors in humans. Several hereditary diseases are characterized by the development of cartilaginous tumors, including hereditary multiple exostoses, metachondromatosis, and enchondromatosis, which are caused by mutations in genes such as *exostosin 1* and *2*, *tyrosine protein phosphatase non-receptor type 11*, *parathyroid hormone receptor 1*, and *isocitrate dehydrogenase 1* and *2*. The proteins encoded by these genes are directly or indirectly linked to fibroblast growth factor (FGF) signaling. In addition, osteochondroma was found in several members of a family with camptodactyly, tall stature, and hearing loss syndrome, a rare inherited disorder caused by a heterozygous missense mutation in *Fgfr3*. In this study, we found that *Fgfr3* deficiency leads to the formation of cartilaginous tumors, including osteochondromas and enchondromas, likely due to dysregulated endochondral ossification in growth plates. We also show that cartilaginous tumorigenesis in *Fgfr3*-deficient mice results from excessive Indian hedgehog production mediated by the activation of mitogen-associated protein kinase signaling. Based on these results, we propose a model for cartilaginous tumor development in which FGFR3 functions as a tumor suppressor. Our findings also suggest that modulation of FGFR3 and related signaling pathways is a potential therapeutic strategy for treating benign cartilaginous tumors.

## Introduction

Enchondromas and osteochondromas are the most frequently occurring benign cartilaginous tumors affecting the skeleton [1,2]. The former develop as ectopic cartilaginous tissue within the bone marrow, while the latter manifest as cartilage-covered bony lesions arising on the bone surface [3]. Both tumor types can potentially undergo malignant transformation to become central or peripheral chondrosarcomas, respectively [4]. Since cartilaginous tumors are resistant to conventional chemo- and radiotherapy, surgical excision is the only treatment option [1]. A deeper understanding of the pathogenic mechanisms underlying cartilaginous tumor development is essential for the development of effective therapeutic strategies.

Cartilaginous tumors arise as a result of mutations in several genes. Hereditary multiple exostoses syndrome (HME, also called hereditary multiple osteochondromas, OMIM 133700) is associated with heterozygous loss-of-function mutations in *exostosin* (*Ext*)*1* or *2*, which encode Golgi-associated glycosyltransferases that mediate the polymerization of heparan sulphate (HS) chains [5,6]. Cells deficient in *Ext1* or *2* fail to synthesize sufficient amounts of HS-rich proteoglycan (HSPG), which is required for the regulation of cell surface and matrix-associated signaling pathways such as Indian hedgehog (IHH), Wnt/β-catenin, fibroblast growth factor (FGF), and bone morphogenetic protein [6]. Although *Ext1/2*-deficient mice develop multiple bony lesions with features similar to osteochondromas in HME patients [7–10], the underlying mechanisms remain unclear. In addition, patients with enchondromatosis (Ollier disease and Maffucci syndrome, OMIM 166000) carry mutations in parathyroid hormone-related protein (PTHrP) receptor (*Pthr*)*1* [11], while mice with the *Pthr1* R150C mutation exhibit constitutive activation of HH signaling and develop multiple enchondroma-like lesions [11,12]. A somatic gain-of-function mutation in the *isocitrate dehydrogenase* (*Idh*)*1/2* gene was also found to induce enchondroma formation in patients with Ollier disease and Maffucci syndrome [13–17].

Recently, the autosomal dominant disease metachondromatosis (MC, OMIM 156250) was found to be associated with heterozygous inactivating mutations in *tyrosine protein phosphatase non-receptor type* (*Ptpn*)*11* [18,19], which encodes the tyrosine phosphatase SHP2, a downstream effector of receptor tyrosine kinases that activates the Ras/extracellular signal-regulated kinase (ERK) pathway [20]. MC is a rare disease characterized by enchondromas and osteochondromas [19]. The role of *Ptpn11* in skeletal development and cartilaginous tumor formation has been investigated in mice [21–24]; interestingly, different mechanisms underlie the development of chondroma-like lesions in *Shp2*-deficient mice, enchondroma-like lesions in *Pthr1* R150C mutant mice, and osteochondroma-like lesions in *Ext1*-deficient mice [24]. Most cartilaginous tumors are formed during skeletal development in a location adjacent to growth plates, suggesting that they arise as a result of dysregulated endochondral bone growth [1]; this is supported by the fact that the majority of the above-mentioned genes are involved in the regulation of growth plate development [24–26].

FGF receptor (FGFR)3 is a transmembrane receptor that regulates skeletal development [27]. Patients with activating FGFR3 mutations exhibit skeletal dysplasias characterized by short stature including achondroplasia (ACH, OMIM 100800), thanatophoric dysplasia I/ II (TD I, OMIM 187600 and TD II,OMIM 187601), hypochondroplasia (OMIM 146000), and severe ACH with developmental delay with acanthosis nigricans (SADDAN, OMIM 187600) [27]. Activated FGFR3 leads to impaired growth plate chondrocyte proliferation and differentiation, resulting in disordered endochondral bone growth and skeletal dysplasia in ACH/TD [28–30]. In contrast, a loss-of-function mutation in *Fgfr3* in humans causes camptodactyly, tall stature, and hearing loss (CATSHL) syndrome (OMIM 610474) [31,32], and *Fgfr3* deletion in mice leads to skeletal overgrowth due to enhanced proliferation of growth plate chondrocytes [33,34]. These data indicate that FGFR3 negatively regulates endochondral bone growth, and that FGFR3 mutations may involve in the development of cartilaginous tumors.

In cartilage, FGFs such as FGF18 require HSPG as a cofactor to bind to the extracellular domain of FGFR3 [35,36]. Intracellular receptor tyrosine kinase domains then recruit the SHP2–growth factor receptor-bound protein 2–son of sevenless 1 signaling complex to the cell membrane via adapter proteins, resulting in the activation of downstream effectors such as mitogen-associated protein kinase (MAPK), AKT, and signal transducer and activator of transcription [27,37,38]. FGFR3 signaling can also act as an inhibitor to regulate a negative feedback loop involving IHH and PTHrP [27]. Thus, most genes implicated in the formation of benign cartilaginous tumors are associated with FGF/FGFR3 signaling. Moreover, Colvin *et al* previously found that some global *Fgfr3* knockout (*Fgfr3* ^*-/-*^) mice develop ectopic pockets of hypertrophic chondrocytes below growth plates [34], and Toydemir *et al* reported that osteochondroma has been detected in the long bones of several members of a family with CATSHL syndrome [31]. However, the precise role of FGFR3 signaling in cartilaginous tumorigenesis is not known. In this study, we demonstrate that conditional *Fgfr3* knockout (*Fgfr3* cKO) mice lacking FGFR3 in chondrocytes exhibit severe enchondroma- and osteochondroma-like lesions in the skeleton, indicating that FGFR3 is critically involved in the formation of cartilaginous tumors.

## Results

### Morphological and histological analysis of the skeleton in *Fgfr3*-deficient mice

**Fig 1:**
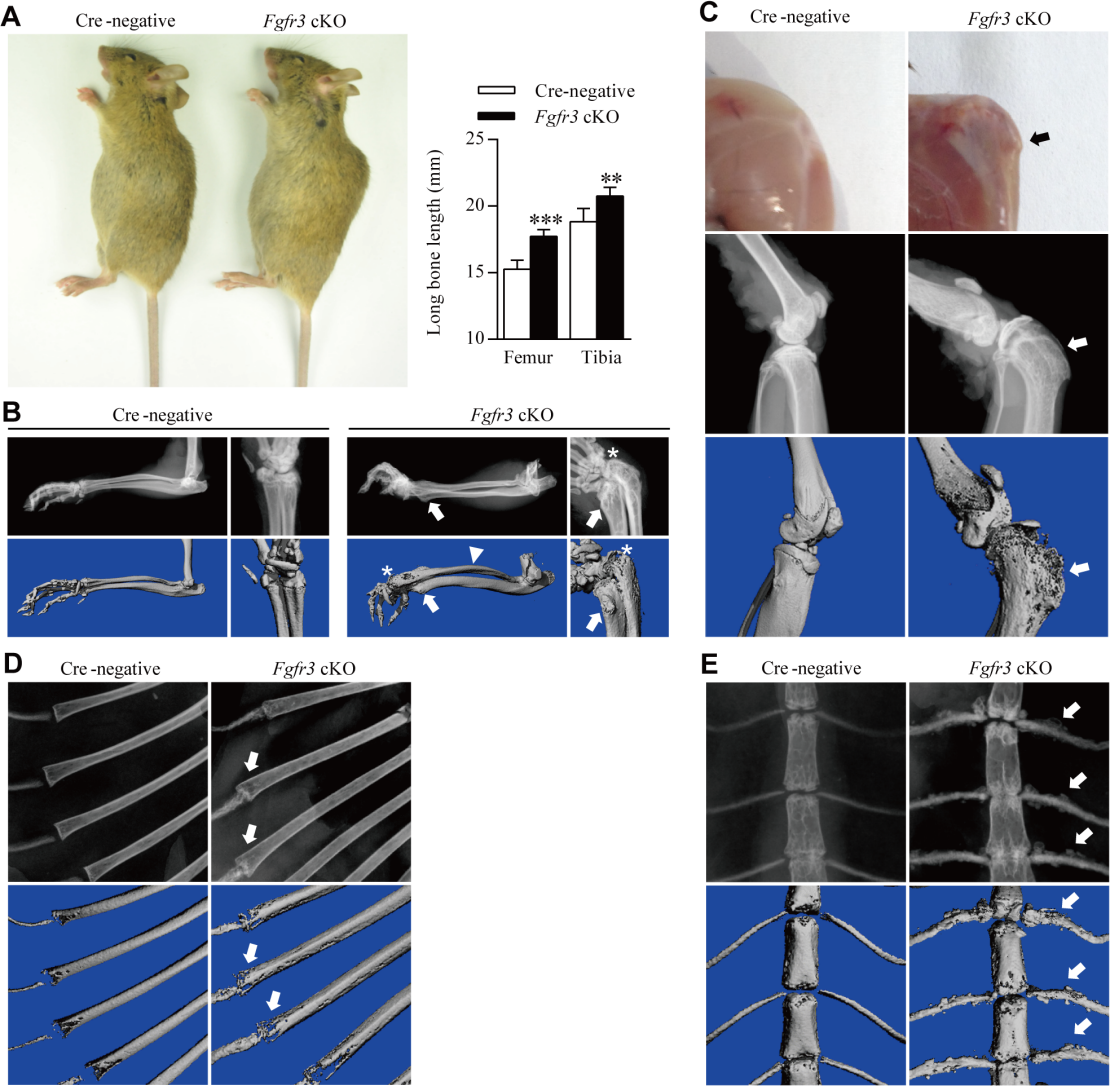
Gross morphology and radiographic assessment of skeletal phenotypes in *Fgfr3* cKO mice. (**A**) Gross morphology of 12-week-old *Fgfr3* cKO mice with large stature. Femur and tibia lengths were determined from X-ray images; their lengths were increased in *Fgfr3* cKO as compared to Cre-negative mice. Values represent mean ± SD. ***p < 0.001, **p < 0.01 (n = 10). (**B**) X-ray and Micro-CT images showing subluxation/dislocation of the radial head (asterisks) and arch-like deformation of the radius (arrowheads) in *Fgfr3* cKO mice. Bony lesions (arrows) in mutants were attached to the distal ulna and radius. (**C**) Gross morphology and X-ray/micro-CT images of the knee joint of 12-week-old *Fgfr3* cKO mice after skin removal showing a bony lesion (arrows) contiguous with the proximal tibia affected by knee joint deformity (arrowheads). (**D, E**) Rib bones showing aberrant radiographic density (D, arrows) and an irregular costal cartilage surface (E, arrows) were observed by X-ray and micro-CT in *Fgfr3* cKO mice.

To investigate the role of FGFR3 in postnatal skeletal growth, we monitored the overall growth of *Fgfr3* cKO relative to *Fgfr3* ^*f/f*^ Cre-negative control mice after tamoxifen administration. *Fgfr3* cKO mice exhibited an increase in body length compared to Cre-negative mice (Fig 1A). Similarly, femur and tibia lengths increased by 13.79% and 9.32%, respectively, in mutants as compared to that in controls. The increased stature of *Fgfr3* cKO mice is consistent previous findings in conventional *Fgfr3* knockout mice and patients with CATSHL syndrome [31–34].

Lesions were observed around the knee joints of 12-week-old *Fgfr3* cKO mice (Fig 1C, upper panels). Since these lesions appeared to be connected with bony tissue, we performed X-ray and micro-computed tomography (CT) analyses of undecalcified bone samples. The X-ray analysis showed dramatically expanded growth plates in the deformed knee joints (Fig 1C, middle panels) The broadly based sessile lesion was located near the growth plate and contained a medulla that was contiguous with the underlying bone (Fig 1C, middle panels). Micro-CT 3-dimensional (3-D) images of *Fgfr3* cKO knee joints showed that the lesion surface formed a non-calcified cap (Fig 1C, lower panels). X-ray and micro-CT scans of 25-week-old *Fgfr3* cKO mice revealed the progression of bony lesions (S1 Fig). We also detected multiple lesions in the wrist, an arch-like deformation of the radius, and subluxation/dislocation of the radial head in mutants (Fig 1B), which also showed heterogeneous radiodensities of costochondral junctions by X-ray (Fig 1D) and multiple bony lesions around the costal cartilage surface (Fig 1E). There were no significant lesions observed in bones with fused growth plates such as digits in these mice during the 12-month observation period (S2A Fig).

**Fig 2:**
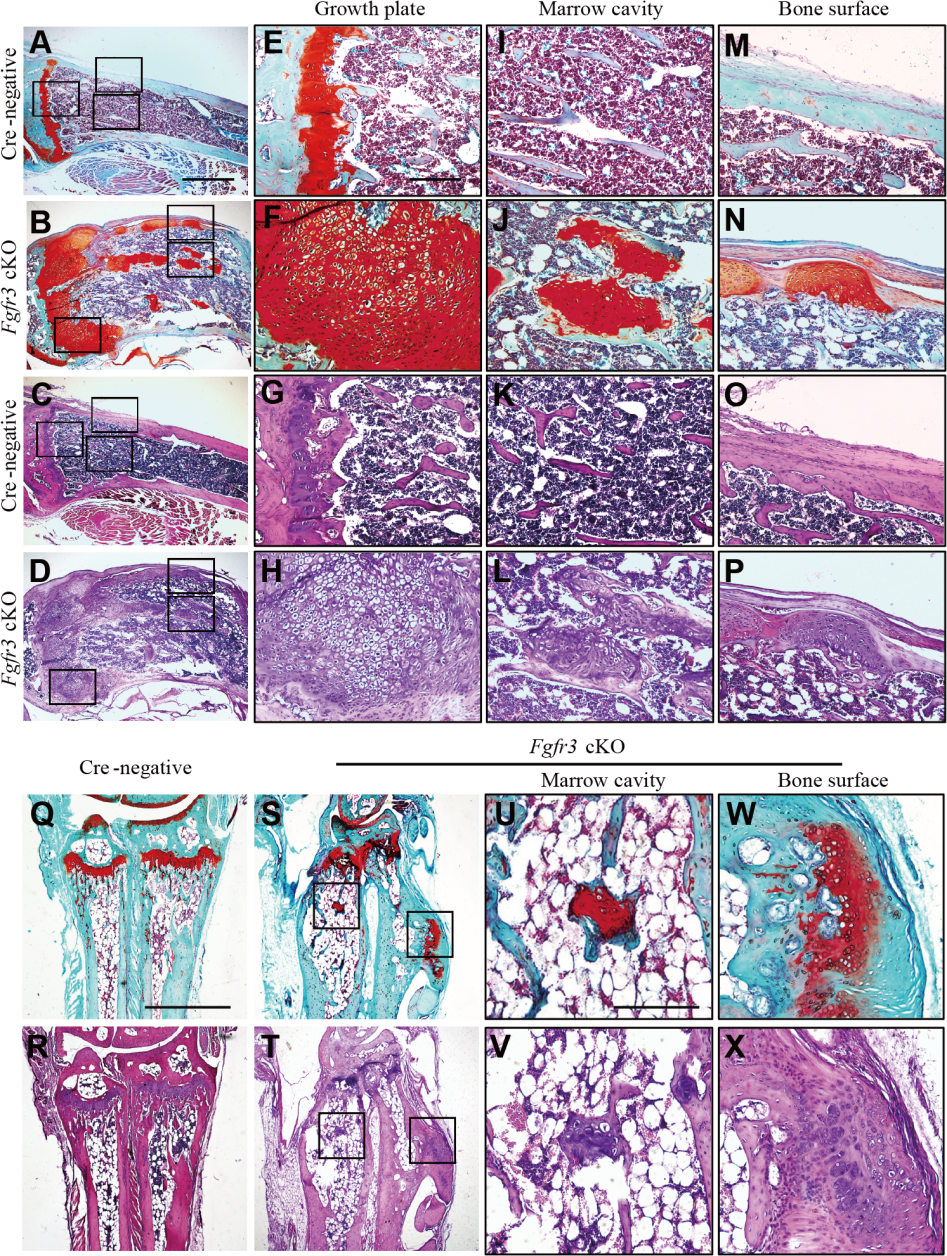
Histological assessment of the knee and wrist in *Fgfr3* cKO mice. (**A**–**P**) Fast green/Safranin O- and H & E-stained sagittal sections of the proximal tibia of Cre-negative and *Fgfr3* cKO mice. (**A**–**D**) Images of multiple osteochondroma- and enchondroma-like lesions around disordered growth plates of the tibia in *Fgfr3* cKO but not in Cre-negative mice. Higher magnification views of areas shown in boxes in E–P. (**E**–**H**) Expansion of the region occupied by hypertrophic chondrocytes in *Fgfr3* cKO growth plates. (**I–L**) Images of enchondroma-like lesions attached to lamellar bone trabeculae in *Fgfr3* cKO mice. (**M–P**) Structure of the cartilaginous cap in *Fgfr3* cKO mice resembling growth plate cartilage. (**Q**–**X**) Fast Green/Safranin O- and H & E-stained coronal sections of the ulna and radius of Cre-negative and *Fgfr3* cKO mice. Higher magnification views of areas shown in boxes in U–X. (**U**–**X**) Images of enchondroma- (U, V) and osteochondroma-like (W, X) lesions in the ulna and radius of *Fgfr3* cKO mice. Scale bar: 1 mm (A–D, Q–T), 200 μm (E–P, U–X).

**Fig 3:**
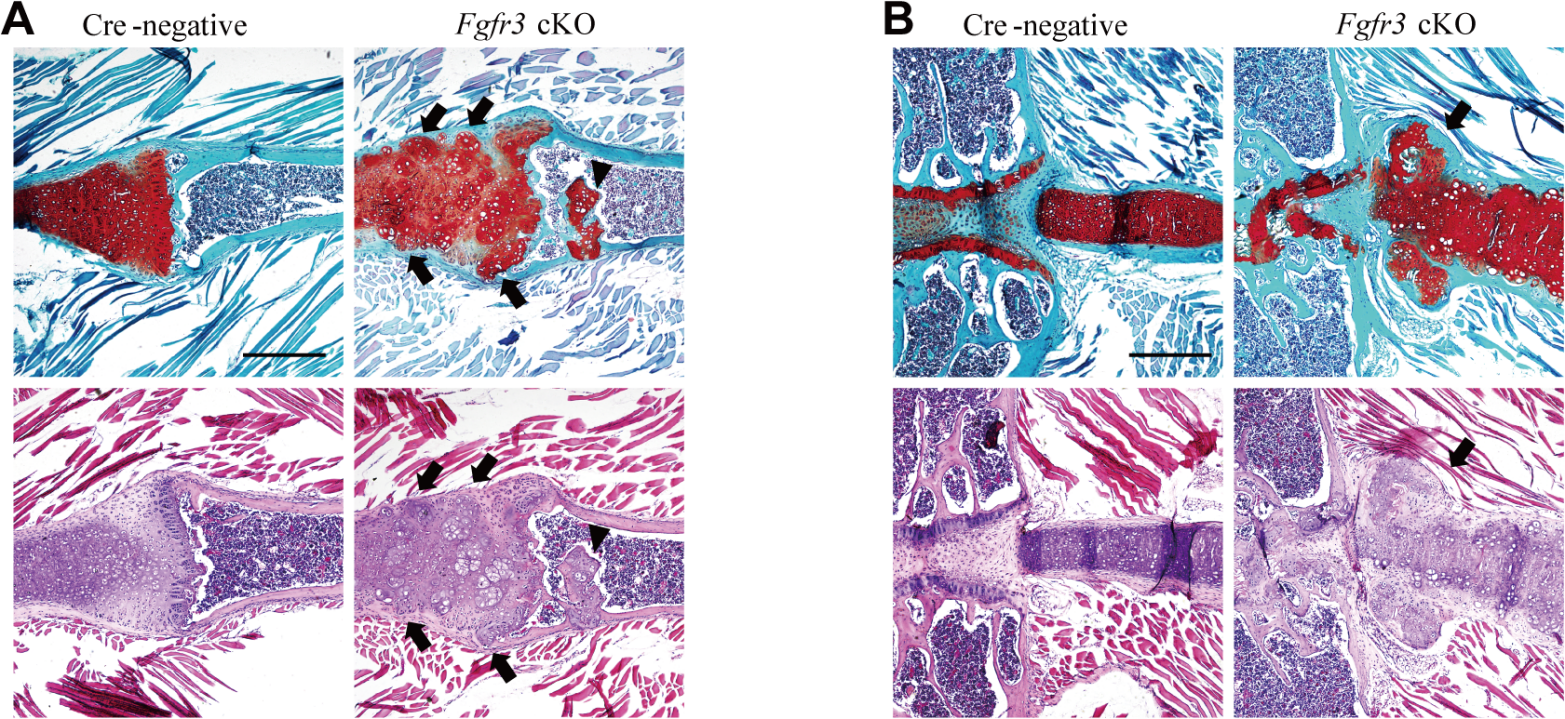
Histological assessment of the rib in *Fgfr3* cKO mice. (**A, B**) Fast Green/Safranin O- and H & E-stained coronal sections of the rib bone from *Fgfr3* cKO mice showing multiple hypertrophic chondrocyte clusters in the costal cartilage. Some clusters (arrows) disrupted the continuity of the perichondrium in the cartilage. Cartilage islands (arrowhead) were observed within the trabecular bone of the rib in *Fgfr3* cKO mice. Scale bar: 400 μm.

Histological analyses were carried out in order to investigate the morphological and cellular changes underlying the skeletal abnormalities in *Fgfr3* cKO mice. Sections of wrist and knee joints from 12-week-old mutant mice showed multiple osteochondroma- and enchondroma-like lesions around the growth plate of the tibia (Fig 2A–2P), femur (Table 1), ulna, and radius (Fig 2Q–2X). Enchondroma-like lesions consisted of cartilaginous lobules with irregular architecture and cellular pleomorphism (Fig 2I–2L and 2U and 2V). Osteochondroma-like lesions that were structurally similar to growth plate cartilage were observed at the bone surface (Fig 2M–2P and 2W and 2X). Notably, growth plates in mutants were expanded and the columnar morphology of chondrocytes was lost (Fig 2E–2H). Multiple hypertrophic chondrocyte clusters were present in the costal cartilage of *Fgfr3* cKO mice (Fig 3A and 3B). In some cases, the expansion of the chondrocyte cluster territory disrupted the integrity of the perichondrium. We also found cartilaginous islands within the trabecular bone of the ribs in mutants.

**Table 1:** Occurrence of chondroma-like lesions in *Fgfr3*-deficient mice.

Analysis of bilateral limb		Occurrence							
		*Fgfr3* ^*f/f*^ ^a^		*Fgfr3* ^*f/f*^; *Col2a1-CreER* ^*T2*^ ^a^		*Fgfr3* ^*f/f*^;*Col2a1-Cre* ^b^		*Fgfr3* ^-/-^ ^b^	
		Osteo-chondroma ^c^	En-chondroma ^d^	Osteo-chondroma ^c^	En-chondroma ^d^	Osteo-chondroma ^c^	En-chondroma ^d^	Osteo-chondroma ^c^	En-chondroma ^d^
Anterior limb	Radius	0/18	0/18	5/22	2/22	2/15	2/15	2/28	2/28
	Ulna	0/18	0/18	4/22	2/22	2/15	1/15	2/28	2/28
Posterior limb	Femur	0/18	0/18	9/22	12/22	3/15	4/15	1/28	2/28
	Tibia	0/18	0/18	14/22	10/22	5/15	3/15	3/28	3/28
Digit		0/18	0/18	0/22	0/22	0/15	0/15	0/28	0/28

^a^ Phenotypes were analyzed at the age of 12 weeks in mice after tamoxifen injection.

^b^ Phenotypes were analyzed at the age of 12 weeks in mice.

^c^ Osteochondroma-like lesion was analyzed from radiographic and histological images.

^d^ Enchondroma-like lesion was analyzed from histological images.

Enchondroma- and osteochondroma-like lesions were observed in several *Fgfr3*-deficient mouse models (Table 1). Although the incidence of chondroma-like lesions in other *Fgfr3*-deficient mice was relatively low, their locations and histological features were similar to what we observed in *Fgfr3* cKO mice (S2B Fig). It is worth noting that those mice with conditional *Fgfr3* deletion in chondrocytes had a higher incidence and greater severity of chondroma-like lesions. To determine whether wild-type chondrocytes contribute to cartilaginous tumorigenesis, cartilage tissues from chondroma-like lesions of *Fgfr3* cKO mice were isolated by laser-capture microdissection (S3A Fig) and analyzed by allele-specific PCR. The lesions contained a mixture of wild-type and *Fgfr3*-deficient cells (S3B Fig); the fraction of *Fgfr3*-deficient cells ranged from 54.94% to 83.9% (S3B Fig). These results suggest that *Fgfr3*-null and wild-type cells interact to promote the formation of cartilaginous tumors, which can explain differences in the incidence and severity of chondroma-like lesions between conditional and global *Fgfr3* knockout mice. A histological analysis revealed ectopic cartilage at the bone-ligament attachment site between the menisco-tibial ligament and meniscus in *Fgfr3* ^*−/−*^ mice (S4E–S4H Fig). This phenotype was similar to that of SHP2 cKO mice in which the *protein tyrosine phosphatase non-receptor type 11* gene was deleted in *fibroblast-specific protein 1-Cre*-expressing fibroblasts [24]. In our study, ectopic cartilage was also observed adjacent to osteochondroma-like lesions connected to the side of growth plate cartilage in *Fgfr3* ^*−/−*^ mice (S4I–S4P Fig); however, we were unable to determine the precise anatomical location due to structural changes caused by the presence of chondroma-like lesions.

### Loss of FGFR3 in mouse chondrocytes disrupts chondrocyte polarity, proliferation, differentiation, and apoptosis in growth plates

**Fig 4:**
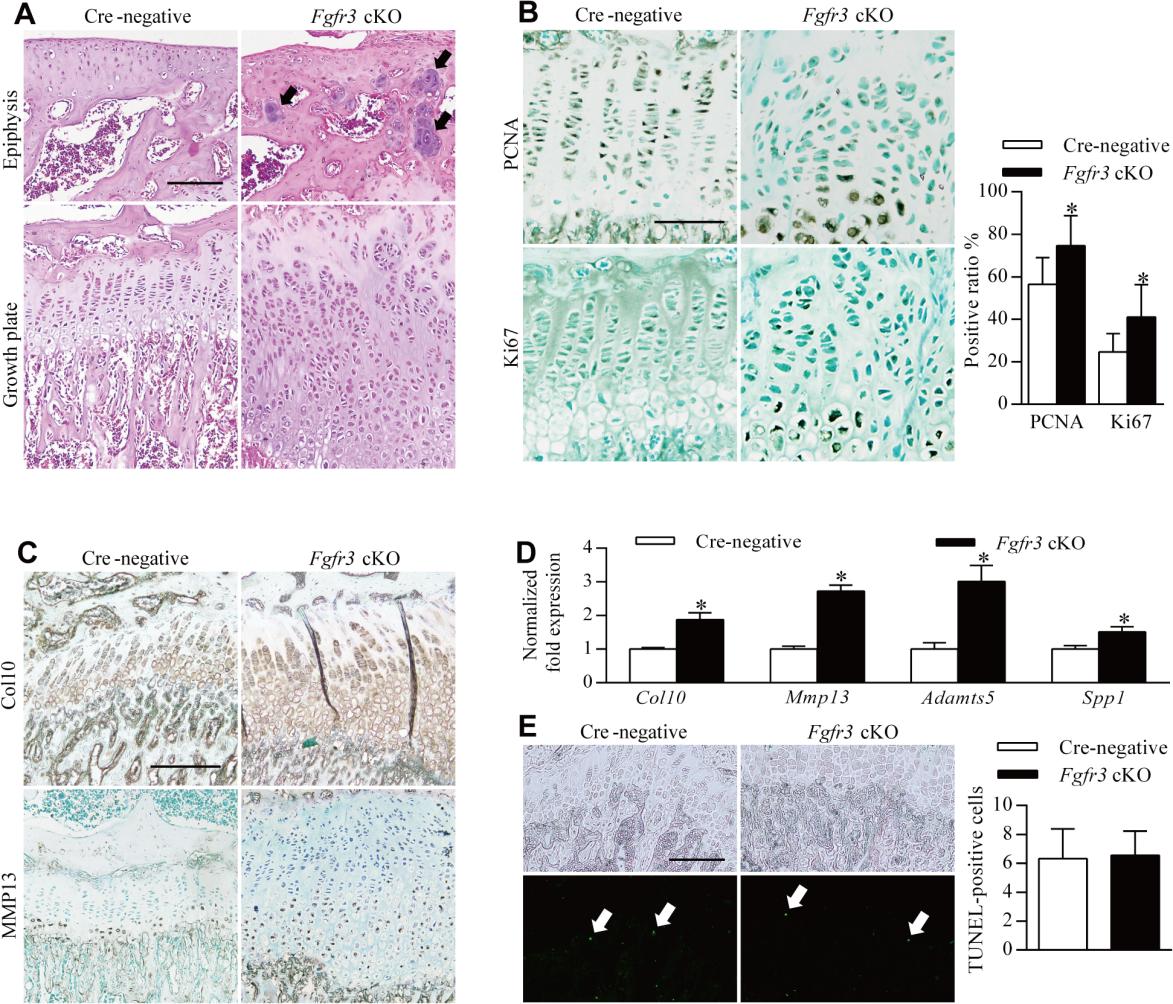
Impaired homeostasis of growth plate chondrocytes in *Fgfr3* cKO mice. Sections of the tibia from 8-week-old mice injected with tamoxifen for 4 weeks were analyzed by histology or immunohistochemistry. (**A**) H & E-stained sagittal sections of the proximal tibia of a Cre-negative (control) mouse showing growth plate chondrocytes organized into columns, in contrast with the expanded and disorganized growth plate of *Fgfr3* cKO mice. Dense chondrocyte clusters (upper panel, arrows) within the epiphysis region were observed in *Fgfr3* cKO mice. (**B**) PCNA- and Ki67-positive cells in the proliferative zone of Cre-negative mice; a greater number of immunoreactive cells were observed in *Fgfr3* cKO mice and were also present in the hypertrophic zone. The ratio of PCNA- to Ki67-positive cells in growth plates was calculated. Values represent mean ± SD. *p < 0.05 (n = 3). (**C**) Expansion of collagen 10 and MMP 13 expression domains in the growth plate of *Fgfr3* cKO mice. (**D**) qRT-PCR analysis of mRNA expression in primary chondrocytes from *Fgfr3* cKO and Cre-negative control mice. Data are expressed as the percent expression relative to controls. Values represent mean ± SD. *p < 0.05 vs. controls. (**E**) Apoptosis in growth plates of Cre-negative and *Fgfr3* cKO mice. Total TUNEL-positive cells (arrows) in the growth plate hypertrophic zone were counted. Values represent mean ± SD (n = 9). Scale bar: 200 μm (A, C, D, E); 100 μm (B).

The observed chondroma-like lesions suggest that loss of *Fgfr3* in chondrocytes at the postnatal stage affects the normal development and maintenance of growth plate cartilage by disrupting the coordination of chondrocyte proliferation, differentiation, and apoptosis [27]. The lesions were associated with the dysregulation of growth plate chondrocytes (Fig 2B and 2D); thus, to determine how *Fgfr3* deficiency alters growth plate development and homeostasis, we performed histological and immunohistochemical analyses on the long bones of 8-week-old mice. As expected, Cre-negative mice exhibited normal growth plate organization in which chondrocytes formed distinct resting, proliferative, and hypertrophic zones (Fig 4A, left panels). In contrast, growth plates in the mutants had disorganized columns of chondrocytes stacked within the proliferative zone, with cell clusters in the resting zone and epiphysis (Fig 4A, right panels), suggesting impaired growth plate polarity. To investigate this possibility, primary cilia were detected using an anti-α-tubulin antibody. A virtual axis oriented parallel to the vertical axis of growth plates was formed by the alignment of primary cilia crossing the center of the chondrocyte column in the proliferative zone of Cre-negative mice (S5A Fig). In contrast, in *Fgfr3* mutants, primary cilia of the aligned axis of chondrocytes in the growth plate were disoriented (S5B Fig). Furthermore, although most chondrocytes in chondroma-like lesions had disrupted polarity, a few formed well-organized columns with an orientation perpendicular to the boundary between the lesion and marrow cavity (S5C and S5D Fig). These data suggest that disruption of chondrocyte polarity may be responsible for the disorganization of growth plates and the formation of chondroma-like lesions in mice with absence of *Fgfr3*.

To determine whether chondrocyte proliferation was altered in growth plates of *Fgfr3* cKO mice, proliferating cell nuclear antigen (PCNA) and Ki-67 expression was detected by immunohistochemistry. PCNA-positive cells were evenly distributed in the proliferative zone of growth plates in control mice (Fig 4B, upper left panel). In contrast, PCNA-(Fig 4B, upper right panel) and Ki67-positive cells (Fig 4B, lower panels) showed an irregular distribution in the proliferative and hypertrophic zones in mutants and had higher rates of proliferation (Fig 4B), consistent with observations from the growth plates of *Fgfr3* ^*−/−*^ mice [33]. Given the expansion of the hypertrophic zone in *Fgfr3*-deficient growth plates (Fig 2E–2H), the expression of collagen 10 and matrix metalloproteinase (MMP)13 was evaluated by immunohistochemistry to determine whether growth plate chondrocyte differentiation was induced in mutants. Immunoreactivity for these markers was increased in *Fgfr3* cKO relative to control mice (Fig 4C). Furthermore, the expression of the terminal differentiation markers *Mmp13*, *a distintegrin and metalloproteinase with thrombospondin motifs* (*Adamts*)*5*, and *secreted phosphoprotein* (*Spp*)*1* [24] was upregulated in *Fgfr3*-deficient chondrocytes (Fig 4D). These data suggest that FGFR3 negatively regulates both proliferation and early/terminal differentiation of chondrocytes in postnatal growth plates.

The role of FGFR3 in chondrocyte apoptosis remains controversial [27]; although FGFR3 overexpression was found to promote chondrocyte apoptosis in vitro [39–42], there is little in vivo evidence to support this observation [43]. We performed terminal deoxynucleotidyl transferase dUTP nick end labeling (TUNEL) to determine whether *Fgfr3* deficiency promotes growth plate chondrocyte apoptosis. In primary chondrocytes isolated from *Fgfr3* cKO mice, the proportion of TUNEL-positive cells was similar to control levels (S7 Fig). In contrast, an in vivo examination found that the proportion of TUNEL-positive cells relative to the total number of hypertrophic chondrocytes was significantly reduced in mutants as compared to control mice (Fig 4E). In addition, the osteoclast activity was considered to be involved in endochondral bone growth [44]. To investigate the potential role of osteoclast activity in pathogenesis of the skeletal phenotypes of *Fgfr3* cKO mice, we performed tartrate-resistant acid phosphatase staining of bone tissue, but found no differences in growth plate osteoclast recruitment between mutant and control mice (S6A Fig). Furthermore, there were no bony lesions detected in osteoclast progenitor-specific *Fgfr3* knockout mice (*Fgfr3* ^*f/f*^; *lysozymeM-Cre*) by radiography (S6B Fig).

To further assess the tumorigenic properties of *Fgfr3*-deficient chondrocytes, we transplanted chondrocytes from *Fgfr3* cKO and control mice subcutaneously into 5-week-old athymic mice. Two of five Cre-negative and four of five *Fgfr3*-deficient cartilage masses were detected and harvested 4 weeks after transplantation, and the *Fgfr3*-deficient cartilage masses were significantly larger after 4 weeks than those derived from Cre-negative cells (S8A and S8B and S8G Fig) and a histological analysis revealed larger areas of matrix-enriched cartilage in the former (S8C–S8F Fig). These data indicate that FGFR3 plays a key role in the postnatal development and homeostasis of growth plate chondrocytes, and that FGFR3 deficiency leads to increased number of disordered chondrocytes, resulting in the formation of chondroma-like lesions.

### MAPK signaling is impaired while IHH expression is upregulated in *Fgfr3* mutants

**Fig 5:**
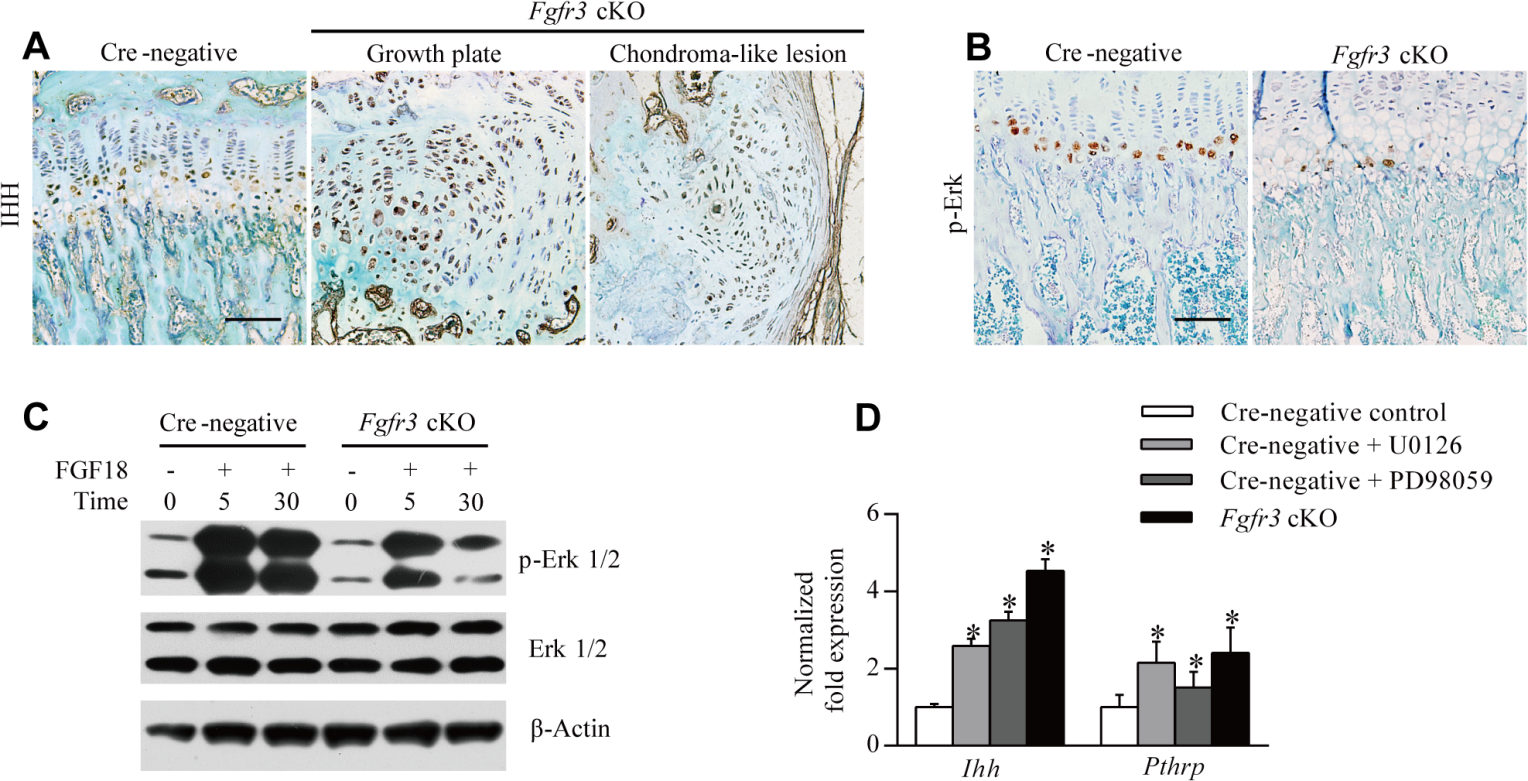
Loss of *Fgfr3* suppresses ERK activation but enhances IHH expression. (**A**) IHH protein expression in growth plates was enhanced in *Fgfr3* cKO relative to Cre-negative mice, and was abundant in chondroma-like lesions of mutants, as determined by immunohistochemistry. (**B**) Phospho-ERK expression was reduced in growth plates of *Fgfr3* cKO mice. (**C**) ERK activity in response to FGF18 was impaired in *Fgfr3*-deficient chondrocytes, as determined by western blotting. (**D**) Control chondrocytes, chondrocytes treated with MEK inhibitor (250 nM U0126 or 10 nM PD98059), and *Fgfr3*-deficient chondrocytes were evaluated for *Ihh* and *Pthrp* expression by qRT-PCR. Data are expressed as the percent expression relative to controls. Values represent mean ± SD. *p < 0.05 vs. controls. Scale bars: 200 μm.

IHH and PTHrP signaling are important for the development and maintenance of growth plate cartilage [45]. Moreover, previous studies have shown that *Pthr1* R150C mutant mice and those overexpressing Gli2, a downstream effector of IHH signaling develop enchondroma-like lesions [11], and we and others have observed that constitutively activated FGFR3 lowers the expression of *Ihh* mRNA in growth plate chondrocytes [28,29,46,47]. We therefore investigated whether IHH signaling is perturbed in the growth plates of *Fgfr3*-deficient mice by immunohistochemistry. In growth plates of Cre-negative mice, IHH expression was mainly detected within the pre-hypertrophic zone and in a few cells in the hypertrophic zone (Fig 5A, left panel). In contrast, the growth plate of *Fgfr3* cKO mice consisted of disorganized chondrocytes in which IHH expression was markedly upregulated (Fig 5A, middle panel). Furthermore, strong IHH expression was observed in chondroma-like lesions in mutants (Fig 5A, right panel), implying that the dysregulation of IHH signaling is associated with cartilaginous tumorigenesis caused by *Fgfr3* deficiency.

It was recently reported that SHP2 deletion in chondrocytes and chondroprogenitor cells within the groove of Ranvier results in ectopic chondrogenesis and the formation of chondroma-like lesions via MAPK-induced upregulation of IHH signaling [23,48]. Given that SHP2 acts as a downstream effector of FGFR3 signaling that modulates the MAPK pathway [27,38], we speculated that cartilaginous tumorigenesis in *Fgfr3* cKO mice was caused by a mechanism similar to that of MC, which results from SHP2 deficiency. To investigate whether MAPK signaling is impaired in chondrocytes of *Fgfr3* cKO mice, we evaluated MAPK activation by immunohistochemistry using an antibody against phosphorylated extracellular signal-regulated kinase (ERK). MAPK activity was detected within hypertrophic zones in Cre-negative growth plates (Fig 5B, left panel), consistent with previous observations [48]. In contrast, MAPK activity was reduced in hypertrophic zones of *Fgfr3* cKO mice (Fig 5B, right panel), which was confirmed by western blot analysis of isolated chondrocytes. MAPK activation in response to FGF18 was also reduced in *Fgfr3*-deficient chondrocytes (Fig 5C). To determine whether the upregulation of IHH is a consequence of impaired MAPK signaling in *Fgfr3* mutants, we examined the expression of genes regulating chondrogenesis by quantitative real-time (qRT-)PCR. *Ihh* and *Pthrp* mRNA levels were increased in *Fgfr3*-deficient chondrocytes (Fig 5D), while treatment with MAPK inhibitor (250 nM U0126 or 10 nM PD98059) also increased *Ihh* and *Pthrp* levels (Fig 5D). These results indicate that the enhanced expression of IHH in growth plates of *Fgfr3* mutants is due at least in part to the suppression of MAPK signaling.

### Inhibiting IHH signaling rescues the skeletal defects of *Fgfr3* mutants

**Fig 6:**
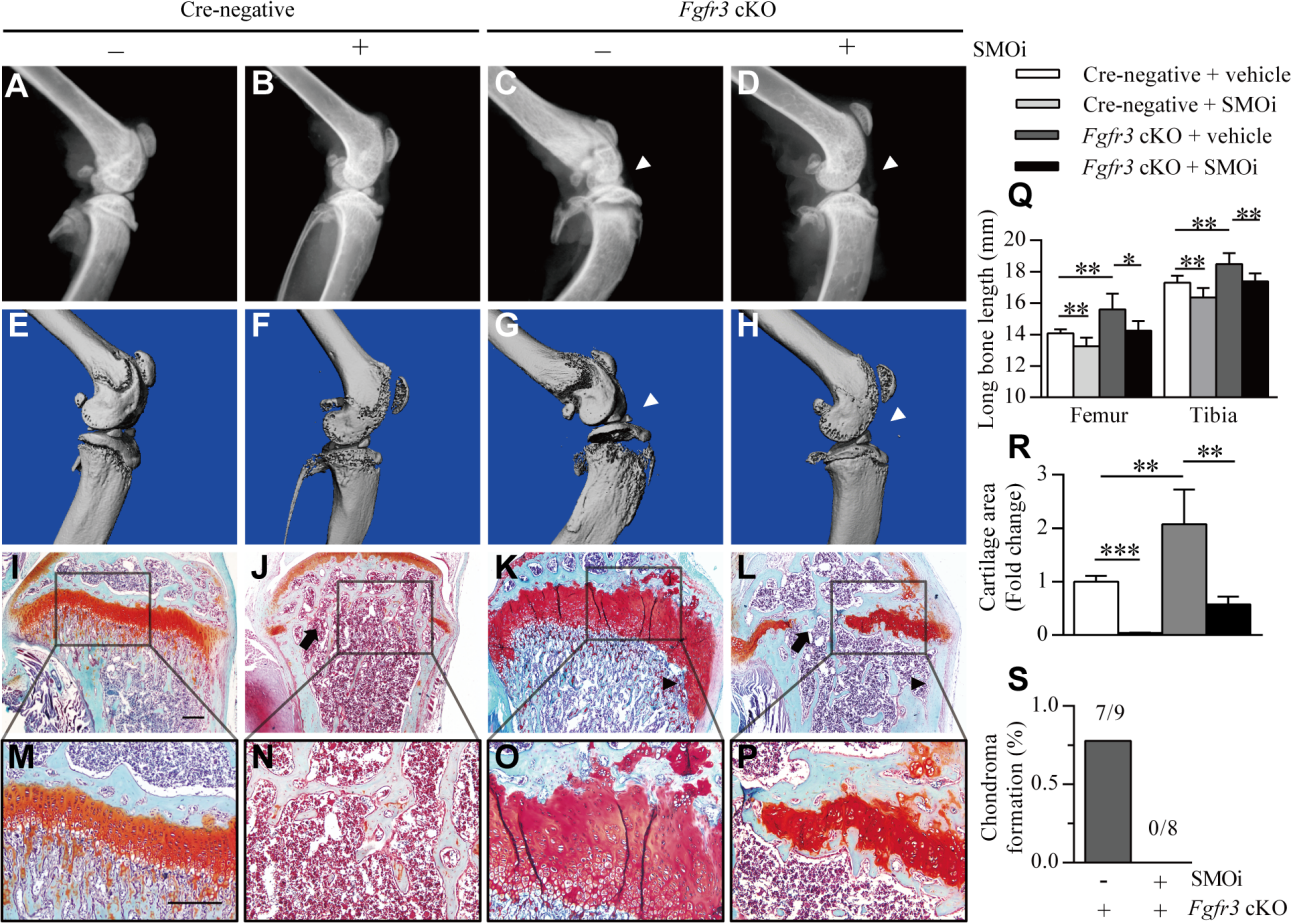
Inhibition of IHH signaling suppresses growth plate expansion and the formation of chondroma-like lesions in *Fgfr3* cKO mice. (**A–H**) X-ray and micro-CT images of knee joints in Cre-negative mice treated with vehicle (n = 9) or SMOi (n = 8) and *Fgfr3* cKO mice treated with vehicle (n = 9) or SMOi (n = 8). SMOi treatment reduced knee joint deformation (white arrowheads) in *Fgfr3* cKO mice. (**I–P**) Fast Green/Safranin O staining showing a reduction in growth plate expansion and chondroma-like lesion formation (black arrowheads) in *Fgfr3* cKO mice after SMOi treatment, which resulted in premature fusion of growth plates (arrows). (**Q**) Femur and tibia length was measured from X-ray images. (**R**) Measurement of the growth plate cartilage area by histomorphometry. (**S**) Chondroma-like lesions of the femur and tibia of *Fgfr3* cKO mice treated with vehicle or SMOi, as visualized by radiographic and histological examination; lesion formation was reduced by SMOi treatment. Values represent mean ± SD. *p < 0.05, **p < 0.01, ***p < 0.001. Scale bar: 200 μm.

In the canonical IHH signaling pathway, Smoothened (Smo) dissociates from the Patched (Ptch) receptor following the binding of IHH to Ptch [49] and activates downstream effectors such as Gli transcription factors [49]. A Smo inhibitor (SMOi) such as GDC-0449 (Vismodegib/Erivedge) developed to treat basal cell carcinoma and medulloblastoma [49,50] can therefore be used to inhibit IHH signaling. Since upregulated IHH signaling is a potential mechanism underlying the cartilaginous tumorigenesis observed in *Fgfr3*-deficient mice, we investigated whether the process could be blocked by inhibiting IHH signaling using GDC-0449. Skeletal phenotypes after SMOi treatment were assessed by X-ray, micro-CT, and histological examination. The lower limb length of all mice was significantly reduced by SMOi treatment (Fig 6Q). X-ray analysis and micro-CT revealed joint deformation and growth plate expansion was detected by histological analyses of *Fgfr3* cKO mice; however, these were alleviated by SMOi treatment (Fig 6A–6P and 6R). Importantly, there were no chondroma-like lesions in the mutants after 4 weeks of SMOi treatment (Fig 6L and 6S). The fusion of growth plates was readily observed in 8-week-old SMOi-treated Cre-negative and *Fgfr3* cKO mice, but not in those receiving vehicle treatment (Fig 6I–6P). Furthermore, SMOi treatment promoted chondrocyte apoptosis in primary chondrocytes isolated from wild-type and mutant mice (S7 Fig). Chondrocytes were evenly distributed at the center of the costal cartilage and were separated from the surrounding muscle by the perichondrium in Cre-negative mice treated either with vehicle or SMOi (S9 Fig). In contrast, hypertrophic-like chondrocyte clusters were observed in the cartilage of *Fgfr3* cKO mice, which were alleviated by SMOi treatment (S9 Fig). These results suggest that enhanced IHH signaling is involved in the pathogenesis of cartilaginous tumorigenesis caused by FGFR3 deficiency.

## Discussion

**Fig 7:**
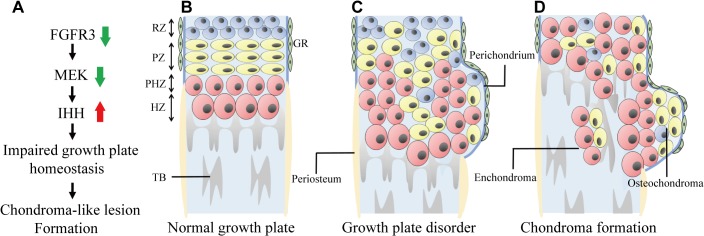
Model of chondroma-like lesion formation in *Fgfr3* cKO mice. (**A**) Effect of *Fgfr3* deficiency on chondroma-like lesion formation. (**B**) Schematic of normal growth plate in long bone. Growth plate chondrocytes are well-organized and surrounded by the perichondrium. (**C**) Loss of *Fgfr3* in growth plate chondrocytes leads to changes in signaling and in chondrocyte polarity, proliferation, differentiation, and apoptosis, resulting in the formation of chondroma-like lesions. (**D**) Growth plate chondrocytes near the perichondrium subsequently form osteochondroma-like lesions, while those in middle region form enchondroma-like lesions. RZ, resting zone; PZ, proliferative zone; PHZ, pre-hypertrophic zone; PZ, hypertrophic zone; TB, trabecular bone; GR, groove of Ranvier.

Cartilaginous tumors are the most common primary bone tumors, with osteochondroma and enchondroma being the most prevalent benign cartilaginous lesions in humans [1]. Mutations in several genes, such as *Ext1/2*, *Ptpn11*, *Pthr1*, and *Idh1/2* lead to cartilaginous tumorigenesis, and these genes are important in the normal development and maintenance of growth plates. Although FGFR3 plays a key role in chondrogenesis, its role in the pathogenesis of cartilaginous tumors is poorly understood; indeed, although osteochondroma is observed in several members of a family with CATSHL syndrome, there have been almost no studies examining this phenotype [31]. One reason for this is that activation of FGFR3 signaling enhances proliferation in most cell types—including fibroblasts, keratinocytes, melanocytes, epithelial cells, lymphocytes and spermatocytes—and consequently lead to cancer [51]; as such, the loss-of-function phenotype of FGFR3—which positively regulates cartilage development and may be responsible for cartilaginous tumorigenesis—may have been previously overlooked. In this study, we found that *Fgfr3* deficiency impaired the normal development and homeostasis of growth plates and induced the formation of chondroma-like lesions via downregulation of MAPK and consequent upregulation of IHH signaling, (Fig 7).

Typical osteochondroma- and enchondroma-like lesions were observed in the femur, tibia, and the distal radius/ulna of *Fgfr3* mutant mice. However, these MC-like phenotypes differ from those of patients with CATSHL syndrome, who exhibit only osteochondroma [31]. A more detailed radiographic examination of these patients is needed in order to determine whether they also have enchondroma lesions.

It remains unclear how chondroma-forming cells are derived. In our study, enchondroma-like lesions in *Fgfr3* cKO mice appeared to originate from disordered growth plates, which is consistent with the enchondroma formation observed in mice with *Shp2* deficiency or overexpression of GLI2 or PTHR1 R150C [11,24,52]. This evidence suggests that enchondroma-forming cells arise from growth plate chondrocytes undergoing abnormal endochondral ossification. The origin of osteochondroma-forming cells is also unknown [6,53]; one possibility is that they result from dysregulated growth plate chondrocytes. Evidence in support of this comes from previous studies showing that *Ext1* deficiency leads to the formation of osteochondroma-like lesions adjacent to growth plate cartilage [7–9,26]. The periosteum and perichondrium both contain chondroprogenitor cells [54,55] and are therefore also considered as potential sites of osteochondroma [10,48]. Other possibilities are that osteochondroma-forming cells are derived from the groove of Ranvier [6,23], or else the periosteum or perichondrium, which showed Cre recombinase activity in the collagen type II α1 (Col2a1)-CreER^T2^ mice used in this study [48]. However, since no ectopic chondrogenesis or chondroma-like lesions was observed in bones with fused growth plates (e.g., adult digits), we speculate that these lesions are mainly induced by the abnormal development and maintenance of growth plate cartilage in *Fgfr3*-deficient mice.

We investigated the roles of FGFR3 in the development and maintenance of growth plate chondrocytes during the postnatal stage to clarify the mechanism by which loss of *Fgfr3* leads to the formation of chondroma-like growth plate lesions. Data from a recent study suggests that *Shp2* deficiency induces the transition from proliferating chondrocytes to pre- or early-hypertrophic chondrocytes but delays the switch from pre- or early hypertrophic to terminal hypertrophic chondrocytes, which is thought to result in chondroma-like lesions in an MC mouse model [24]. In our study, *Fgfr3* deficiency induced both early and terminal differentiation of growth plate chondrocytes in postnatal stages. We therefore propose that different mechanisms are responsible for the formation of chondroma-like lesions in *Fgfr3*- and *Shp2*-deficient mice. The chondroma-like lesions in the former are composed of small chondrocytes surrounded by large hypertrophic chondrocytes and are located in the marrow cavity and at the bone surface adjacent to growth plates. We also observed dysregulated polarity, increased proliferation, and decreased apoptosis of growth plate chondrocytes in *Fgfr3* mutants. Based on these findings, we speculate that *Fgfr3* deficiency in growth plates disrupts the coordination of chondrocyte proliferation, differentiation, and apoptosis, resulting in the accumulation of abnormal chondrocytes that form lesions. Additionally, decreased osteoclast activity and vascular invasion are thought to underlie cartilaginous tumorigenesis [56]. We found that osteoclast/chondroclast recruitment around growth plates and chondroma-like lesions was unaffected in *Fgfr3* cKO mice and there were no chondroma-like lesions in *Fgfr3* ^*f/f*^; *lysozyme (lys)M-Cre* mice during 6 months of observation. Moreover, it was reported that despite the downregulation of vascular endothelial growth factor—a molecule required for hypertrophic chondrocyte apoptosis [57]—vascular invasion is only slightly impaired in the growth plate cartilage of *Fgfr3* ^−/−^ mice [43]. Therefore, changes in osteoclast/chondroclast and vascular invasion may not be involved in chondroma-like lesion formation resulting from loss of *Fgfr3*.

The results of this and previous studies suggest that FGFR3 signaling promotes early chondrocyte hypertrophy through SHP2 and MAPK signaling [24,27]. However, the roles of FGFR3 and ERK1/2 in terminal hypertrophy are different from that of SHP2 in growth plate chondrocytes during the postnatal stage. Although the downregulation of FGFR3, SHP2, and ERK1/2 observed in vivo induced the expansion of the growth plate hypertrophic zone, an increase in bone length was only observed in *Fgfr3*- and *Erk1/2*-deficient mice [22,24,33,34,58]. Moreover, loss of ERK1/2 strongly inhibited chondrocyte proliferation, implying that enhancement of chondrocyte terminal hypertrophy is a major determinant of longitudinal bone overgrowth in *Erk1/2*-deficient mice [58,59]. These studies suggest that ERK1/2 negatively regulates growth plate chondrocyte terminal differentiation during the postnatal stage. Moreover, expression of constitutively active mitoggen-activated protein kinase kinase (MEK)1 can rescue the overgrowth of bones in *Fgfr3*-deficient mice by inhibiting chondrocyte differentiation [60], indicating that the MAPK pathway is activated downstream of FGFR3 signaling to regulate growth plate chondrocyte differentiation. An important remaining question is whether FGFR3 and SHP2 have MAPK-independent and-dependent roles in cartilaginous tumorigenesis.

We also speculate that cell autonomous as well as nonautonomous mechanisms are involved in the initiation and progression of cartilaginous tumors. This is supported by the fact that a recombination rate of only 6%–15% is sufficient to induce the formation of osteochondroma-like lesions in *Ext1* ^*f/f*^ mice, in which osteochondroma-like lesions contain both *Ext1*-null and wild-type chondrocytes [9]. A similar phenomenon was observed in patients with active *Idh1* mutations and in *Shp2*-deficient mice [16,24], suggesting that altered paracrine signaling from mutant growth plate chondrocytes may alter homeostasis in neighboring wild-type cells. In *Fgfr3* ^*−/−*^ mice, FGFR3 is deleted in all cells, and each zone within the growth plate—especially the hypertrophic zone—is expanded [33,34]; however, the phenotype is milder than that of *Fgfr3* cKO mice with mosaic *Fgfr3* deletion. Moreover, the incidence of chondroma-like lesions is much lower in *Fgfr3* ^*−/−*^ than in *Fgfr3* cKO mice (Table 1). One explanation for this is that chondroma formation can be initiated by *Fgfr3*-deficient cells in both mutants, but will be enhanced in mosaic *Fgfr3* cKO mice owing to crosstalk between mutant and adjacent wild-type cells, which are not passively incorporated into lesions but may interact with mutant cells to actively promote cartilaginous tumor formation.

Previous studies have suggested that IHH upregulation contributes to metachondromatosis and that inhibiting IHH signaling suppresses cartilaginous tumorigenesis in *Shp2*-deficient mice [23,48]. Although we found that treatment with an IHH signaling inhibitor reduced the formation of chondroma-like lesions in *Fgfr3* cKO mice, adverse secondary effects were induced, including premature growth plate fusion leading to a shorter long bone, which is similar to what was observed in mice with chondrocyte-specific deletion of IHH [61]. Other types of SMOi such as HhAntag had similar effects on the skeleton [62]. Thus, given that cartilaginous tumors occur mostly in children, caution is required when using SMOi to treat young patients with enchondroma and osteochondroma. PF-04449913 was shown to alleviate cartilaginous tumors in *Shp2*-deficient mice without significantly affecting bone development [23]. However, the optimal timing and dosage of IHH signaling inhibitors such as PF-04449913 must be established in order to improve treatment outcome and minimize the deleterious effects on the developing growth plate.

In conclusion, this first is the first study to demonstrate that loss of *Fgfr3* leads to the downregulation of MAPK signaling and enhanced IHH expression, resulting in the formation of chondroma-like lesions, including enchondromas and osteochondromas. Based on these findings, we propose that FGFR3 has a tumor suppressor-like function in cartilage development. *Fgfr3* cKO mice can thus serve as a model to dissect the roles and mechanisms of action of FGFR3 in cartilaginous tumorigenesis, which can facilitate the development of effective therapies.

## Materials and Methods

### Animals

*Fgfr3* ^*f/f*^ mice were previously generated by our group [63]. The *Col2a1-CreER* ^*T2*^ [64], *Col2al-Cre* [65], *lysM-Cre* (Jackson Laboratories, Bar Harbor, ME, USA), *cytomegalovirus (CMV)-Cre* [66], and *Fgfr3* ^*−/−*^ [33] mice were genotyped as previously described. For inducible deletion of *Fgfr3* in chondrocytes, *Fgfr3* ^*f/f*^ mice were crossed with *Col2a1-CreER* ^*T2*^ mice to obtain *Fgfr3* ^*f/+*^; *Col2a1-CreER* ^*T2*^ mice, which were crossed with *Fgfr3* ^*f/f*^ mice to obtain *Fgfr3* ^*f/f*^ and *Fgfr3* ^*f/+*^; *Col2a1-CreER* ^*T2*^ mice. *Fgfr3* ^*f/f*^ mice were crossed with *Fgfr3* ^*f/+*^; *Col2a1-CreER* ^*T2*^ mice to obtain *Fgfr3* ^*f/+*^; *Col2a1-CreER* ^*T2*^ (*Fgfr3* cKO) and *Fgfr3* ^*f/f*^ (Cre-negative) mice. Tamoxifen (1 mg/10 g body weight) was administered by intraperitoneal (i.p.) injection twice weekly for 8 weeks (for a total of 16 injections) starting 4 weeks after birth. Cre recombinase activity in the postnatal growth plate was evaluated as previously described [48]. As for *Fgfr3* ^*f/+*^; *Col2a1-CreER* ^*T2*^ mice, *Fgfr3* ^*f/f*^ mice were crossed with *Col2al-Cre* and *lysM-Cre* mice to generate *Fgfr3* ^*f/f*^; *Col2al-Cre* and *Fgfr3* ^*f/f*^; *lysM-Cre* mice, respectively. Wild-type mice were mated with the *Fgfr3* ^*f/f*^; *CMV-Cre* mice to generate *Fgfr3* heterozygous mice which are expected to give 50% recombined *Fgfr3* allele. *Fgfr3* ^*+/-*^ mice were mated with the *Fgfr3* ^*+/-*^ mice to generate *Fgfr3* ^*+/+*^ and *Fgfr3* ^*-/-*^ mice. All mice were of the C3H/HeJ background. Animal experiments were performed according to protocols approved by the Laboratory Animal Welfare and Ethics Committee of the Third Military Medical University (Chongqing, China).

### X-ray and micro-CT analysis

X-ray images of bony tissue were obtained using an MX-20 Cabinet X-ray system (Faxitron X-Ray, Tucson, AZ, USA). Each undecalcified specimen was scanned using a vivaCT 40 micro-CT system (Scanco Medical, Brüttisellen, Switzerland). Serial 12.5-μm 2-D and 3-D images were acquired at 70 kV and 113 mA. Constant thresholds (200) were applied to grayscale images to distinguish bone from soft tissue.

### Histological assessment

Samples were fixed in 4% paraformaldehyde in 0.1 M phosphate buffer overnight, decalcified in 15% EDTA-phosphate buffered saline for 2 weeks and embedded in paraffin. Sections (5-μm thick) were stained with Safranin O/Fast Green and hematoxylin and eosin (H & E). For histomorphometric analysis, the Safranin O-positive cartilage area in 5-μm serial sections from the midsagittal region of growth plates was quantified using Image-Pro Plus 5.1 (Leeds Precision Instruments, Minneapolis, MN, USA).

### Immunostaining analysis

Decalcified bone sections were deparaffinized with xylene, and endogenous peroxidase activity was quenched by treatment with 3% H_2_O_2_ for 15 min, followed by antigen retrieval by trypsinization for 10 min. Sections were then blocked with normal goat serum for 30 min and incubated at 4°C overnight with primary antibody followed by the appropriate biotinylated secondary antibody and horseradish peroxidase-conjugated streptavidin-biotin staining. Immunoreactivity was visualized with a 3,3'-diaminobenzidine tetrahydrochloride kit (ZSGB-BIO, Beijing, China) followed by counterstaining with Methyl Green. Primary antibodies against the following proteins were used: PCNA (1:200; BioVision, Milpitas, CA, USA), Ki67 (1:100; Abcam, Cambridge, MA, USA), Col10a1 (1:200; Abcam), MMP13 (1:200; Abcam), phospho-ERK (1:100; Cell Signaling Technology, Danvers, MA, USA), acetylated tubulin (1:200; Sigma-Aldrich, St. Louis, MO, USA), and IHH (1:100; Abcam). The number of PCNA- and Ki67-positive nuclei in three central regions of the growth plate was counted in Cre-negative and *Fgfr3* cKO mice (n = 3 each).

### Primary chondrocyte cultures

Primary chondrocytes were isolated from knee joint cartilage of 3-day-old mice. Dissected tissues with cartilage were first digested with 0.25% trypsinase (Gibco/Life Technologies, Carlsbad, CA, USA) at 37°C for 15 min to remove muscles, ligaments, and bone tissue. Chondrocytes were isolated from knee joints by additional digestion with 0.1% collagenase II (Gibco/Life Technologies) overnight at 37°C in a CO_2_ incubator. Cells were seeded in 12-well plates at a density of 2 × 10^5^ cells/well and cultured in Dulbecco’s Modified Eagle’s Medium/F12 (1:1) supplemented with penicillin/streptomycin (Gibco/Life Technologies) and 10% fetal bovine serum until they reached sub-confluence. On day 3 of culturing, primary chondrocytes were treated with 1 μM 4OH-tamoxifen (Sigma-Aldrich) for 48 h. For MEK inhibitor treatment, chondrocytes were incubated in 250 nM U0126 or 10 nM PD98059 (both from Merck, Kenilworth, NJ, USA) for 24 h after treatment with and in the presence of 4OH-tamoxifen. For FGF18 treatment, 50 ng/ml FGF18 (PeproTech, Rocky Hill, NJ, USA) were added to cultures 5 and 30 min after 4OH-tamoxifen incubation.

### Allotransplantation assay

Primary chondrocytes (2 × 10^6^cells) were resuspended in 100 μl serum-free medium and mixed with an equal volume of Matrigel (BD Biosciences, Franklin Lakes, NJ, USA). The mixture was injected subcutaneously into the lower flanks of athymic nude mice (BALB/c-nu, male, 5 weeks old). Tamoxifen was administered by i.p. injection twice weekly. Chondrocyte transplants were harvested 4 weeks after transplantation.

### qRT-PCR

Total RNA was extracted from primary chondrocytes with TRIzol reagent (Invitrogen, Carlsbad, CA, USA) according to the manufacturer’s instructions. All reactions were performed in a Mx3000P thermal cycler (Stratagene, Santa Clara, CA, USA) using the Two-Step QuantiTect SYBR Green RT-PCR Kit (Takara Biotechnology, Otsu, Japan) and reaction conditions were optimized for each gene by altering the annealing temperature (57°C–61°C). Each run consisted of samples for genes of interest and cyclophilin A. The forward and reverse primer sequences were as follows: *cyclophilin A*, 5'-CGA GCT CTG AGC ACT GGA GA-3' and 5'-TGG CGT GTA AAG TCA CCA CC-3'; *Col10a1*, 5'-GCA GCA TTA CGA CCC AAG AT-3' and 5'-CAT GAT TGC ACT CCC TGA AG-3'; *Mmp13*, 5'-CAG TTG ACA GGC TCC GAG AA-3' and 5'-CGT GTG CCA GAA GAC CAG AA-3'; *Adamts5*, 5'-GGA GCG AGG CCA TTT ACA AC-3' and 5'-CGT AGA CAA GGT AGC CCA CTT T-3'; *Ihh*, 5'-CAA TCC CGA CAT CAT CTT CA-3' and 5'-GCG GCC CTC ATA GTG TAA AG-3'; *Spp1*, 5'-TGG CTA TAG GAT CTG GGT GC-3' and 5'-TTG GCA GTA ATT TGC TTT TG-3'; and *Pthrp*, 5'-CAT CAG CTA CTG CAT GAC AAG G-3' and 5'-GGT GGT TTT TGG TGT TGG GAG-3'.

### Laser capture microdissection and PCR

Tissue sections (10 μm thick) were transferred to polyethylene tetraphthalate FrameSlides (Leica, Wetzlar, Germany) and stained with H & E. Cartilage tissue in the region of chondroma-like lesions was isolated using a laser-capture microdissection system (Molecular Machines & Industries, Eching, Germany). To avoid contamination from cells of the perichondrium/periosteum or trabecular bone, the tissue was cut far away from the border between the chondroma-like lesion and surrounding tissue. DNA was extracted from cartilage tissue using the EZNA DNA extraction kit (Omega Bio-Tek, Norcross, GA, USA) and analyzed by semiquantitative PCR. The p3 and p5 primers were used to detect the unrecombined allele, whereas p1 and p5 were used to detect the recombined allele as previously described [63]. Band intensity was measured using Image Lab software (Bio-Rad Laboratories, Hercules, CA, USA). The ratio of the recombined to unrecombined allele was normalized to the level of *Fgfr3* expression in heterozygous mice (taken as 1:1).

### Western blot analysis

Chondrocytes lysates were prepared in ice-cold radioimmunoprecipitation assay buffer containing protease inhibitors (Roche Applied Science). Proteins were resolved by 10% or 12% sodium dodecyl sulfate polyacrylamide gel electrophoresis and transferred to a polyvinylidene difluoride membrane (Millipore, Billerica, MA, USA), which was probed with antibodies against the following proteins: phospho-ERK (Cell Signaling Technology, Danvers, MA, USA), ERK (Cell Signaling Technology), and β-actin (Sigma-Aldrich). Immunoreactivity was detected by enhanced chemiluminescence (Pierce, Rockford, IL, USA).

### Inhibition of Ihh signaling

For in vitro experiments, the SMOi GDC-0449 (Selleck Chemicals, Houston, TX, USA) was reconstituted in dimethyl sulfoxide (Sigma-Aldrich) and applied at a final concentration of 1 μM for 24 h. For in vivo experiments, GDC-0449 was reconstituted in 50% (w/v) 2-hydroxypropyl-β-cyclodextrin (Sigma-Aldrich) in water. Mice were injected twice weekly for 4 weeks with tamoxifen (1 mg/10 g body weight) starting from 4 weeks of age and daily with GDC-0449 (1 mg/10 g body weight) or vehicle (50% 2-hydroxypropyl-β-cyclodextrin), except on tamoxifen injection days. At 8 weeks of age, the tibia and femur were dissected for X-ray, micro-CT, and histological analyses.

### Apoptosis assay

The TUNEL assay was carried out with the In Situ Cell Death Detection kit (Roche Applied Science, Pleasanton, CA, USA) according to the manufacturer’s instructions.

### Statistical analysis

Results are expressed as mean ± SD. Differences between groups were analyzed with the Student’s t test. p < 0.05 was considered significant.

## Supporting Information

S1 Fig Progression of bony lesions in 25-week-old *Fgfr3* cKO mice. Mice were administered tamoxifen for 8 weeks starting at 4 weeks old and were sacrificed at week 25. X-ray and micro-CT images showing a deformed knee joint (arrowheads) and multiple bony outgrowths (arrows) located near the growth plate in *Fgfr3* cKO mice.
(TIF)

S2 Fig Radiographic assessment of skeletal phenotypes in *Fgfr3*-deficient mice. (**A**) X-ray images of 12-month-old Cre-negative and *Fgfr3* cKO mice. Bony lesions were observed around the growth plate of the tibia, femur, ulna, and radius (arrowheads) in mutants. (**B**) X-ray images of 12-week-old Cre-negative, *Fgfr3* ^*f/f*^; *Col2-Cre*, and *Fgfr3* ^*−/−*^ mice. Multiple bony lesions (arrowheads) were observed in the mutants.
(TIF)

S3 Fig Chondroma-like lesions contain both wild-type and *Fgfr3*-deficient chondrocytes. (**A**) Cartilage tissue of chondroma-like lesions in the tibia isolated by laser-capture microdissection. (**B**) PCR analysis of DNA from chondroma-like lesions in *Fgfr3* cKO mice. DNA from the cartilage of *Fgfr3* heterozygous and Cre-negative mice were analyzed as a control. Chondroma-like lesions in *Fgfr3* cKO mice contained recombined and unrecombined *Fgfr3* alleles; the percentage of recombined *Fgfr3* alleles was determined by semiquantitative PCR in chondroma-like lesions of *Fgfr3* cKO mice of similar phenotypic severity (n = 6). Recombined *Fgfr3* allele = 390 bp; unrecombined *Fgfr3* allele = 320 bp; wild-type allele = 260 bp. Scale bar: 200 μm.
(TIF)

S4 Fig Global knockout of *Fgfr3* induces chondroma-like lesions and ectopic cartilage formation. Tissue sections of tibia were stained with Fast Green/Safranin O and by H & E. (**A**–**D**) Ectopic cartilage and chondroma-like lesions are observed in *Fgfr3* ^*−/−*^ but not in *Fgfr3* ^*+/+*^ mice. Higher magnification views of the areas shown in boxes in E–T. (**E–H**) Ectopic cartilage at the bone-ligament attachment site (BLAS) of the meniscus (M) and menisco-tibial ligament (MTL) in *Fgfr3* ^*−/−*^ mice. (**I–P**) Osteochondroma-like lesion structurally similar to growth plate cartilage adjacent to ectopic cartilage (arrows) in *Fgfr3* ^*−/−*^ mice. (**Q–T**) Enchondroma-like lesion (arrows) embedded in the trabecular bone of *Fgfr3* ^*−/−*^ mice. Scale bar: 1 mm (A–D), 200 μm (E–T).
(TIF)

S5 Fig Organization of chondrocyte primary cilia in the growth plate and chondroma-like lesions in *Fgfr3* cKO mice. (**A**) Primary cilia (green line) were observed on the chondrocyte surface extending into the extracellular matrix of cartilage while those in the proliferative zone were parallel to the vertical axis of the growth plate in Cre-negative mice. (**B**) Primary cilia in the growth plate cartilage of *Fgfr3* cKO mice were misoriented. (**C, D**) Primary cilia of most chondrocytes in chondroma-like lesions were misoriented; some cells were organized into small columns (arrowheads) oriented perpendicularly to the boundary (broken line) between the chondroma-like lesion and marrow cavity (MC). Higher magnification view of the areas shown in boxes in D. Arrow-bar = vertical axis of the growth plate (A, B) or chondroma-like lesion (C, D). Scale bar: 25 μm.
(TIF)

S6 Fig (A) Tartrate-resistant acid phosphatase staining of growth plates from the tibia of 8-week-old Cre-negative and *Fgfr3* cKO mice. Osteoclast recruitment (arrows) around growth plates (GP) and chondroma-like lesions (asterisks) was unaffected in *Fgfr3* cKO mice. (**B**) X-ray images of 6-month-old Cre-negative and *Fgfr3* ^*f/f*^; *lysM-Cre* mice. There was no joint deformation or bony lesions observed in *Fgfr3* ^*f/f*^; *lysM-Cre* mice. Scale bar; 200 μm (A).
(TIF)

S7 Fig SMOi induces apoptosis in Cre-negative and *Fgfr3*-deficient primary chondrocytes. Apoptosis in primary chondrocytes from Cre-negative mice treated with vehicle (dimethyl sulfoxide) or SMOi (GDC-0449) and *Fgfr3* cKO mice treated with vehicle or SMOi was detected by TUNEL. Nuclei were visualized by DAPI staining. TUNEL-positive cells (arrows) in primary chondrocytes were counted and are expressed as a percentage of the total number of cells. Values represent mean ± SD. *p < 0.05, ***p < 0.001 (n = 3).
(TIF)

S8 Fig (A–F) Gross morphology and histological analysis of *Fgfr3*-deficient chondrocyte transplants recovered from recipient mice. Higher magnification views of areas shown in boxes E and F. Fast Green/Safranin O staining showing a larger area of matrix-enriched chondrocytes in *Fgfr3*-deficient as compared to Cre-negative chondrocyte transplants. (**G**) Scatter plots of the volume of recovered chondrocyte transplants. Scale bar: 1 mm (A–D), 200 μm (E, F).
(TIF)

S9 Fig Decreased formation of hypertrophic chondrocyte clusters in the costal cartilage of *Fgfr3* cKO mice following SMOi treatment. Fast Green/Safranin O staining of the costal cartilage in Cre-negative mice treated with vehicle (n = 9) or SMOi (n = 8) and *Fgfr3* cKO mice treated with vehicle (n = 9) or SMOi (n = 8). (**A, B, E, F**) In Cre-negative mice treated with vehicle or SMOi, the costal cartilage is separated from the surrounding muscle by a line of perichondrium. (**C, D, G, H**) Hypertrophic chondrocyte clusters (arrows) disrupt perichondrium organization in *Fgfr3* cKO mice, this phenotype that was attenuated by SMOi treatment. (**I, J, M, N**) Cre-negative mice treated with vehicle or SMOi had chondrocytes that were evenly distributed in the middle of the costal cartilage. (**K, L, O, P**) Formation of hypertrophic chondrocyte clusters (arrows) in the middle of the costal cartilage in *Fgfr3* cKO mice was also reduced by SMOi treatment. Scale bar: 400 μm (A–D, I–L), 100 μm (E–H, M–P).
(TIF)
