# Supplementary figures and images for: FGFR3 Deficiency Causes Multiple Chondroma-like Lesions by Upregulating Hedgehog Signaling
Source: PLoS Genet. 2015 Jun 19;11(6):e1005214. doi: 10.1371/journal.pgen.1005214 (PMC4474636; doi:10.1371/journal.pgen.1005214)

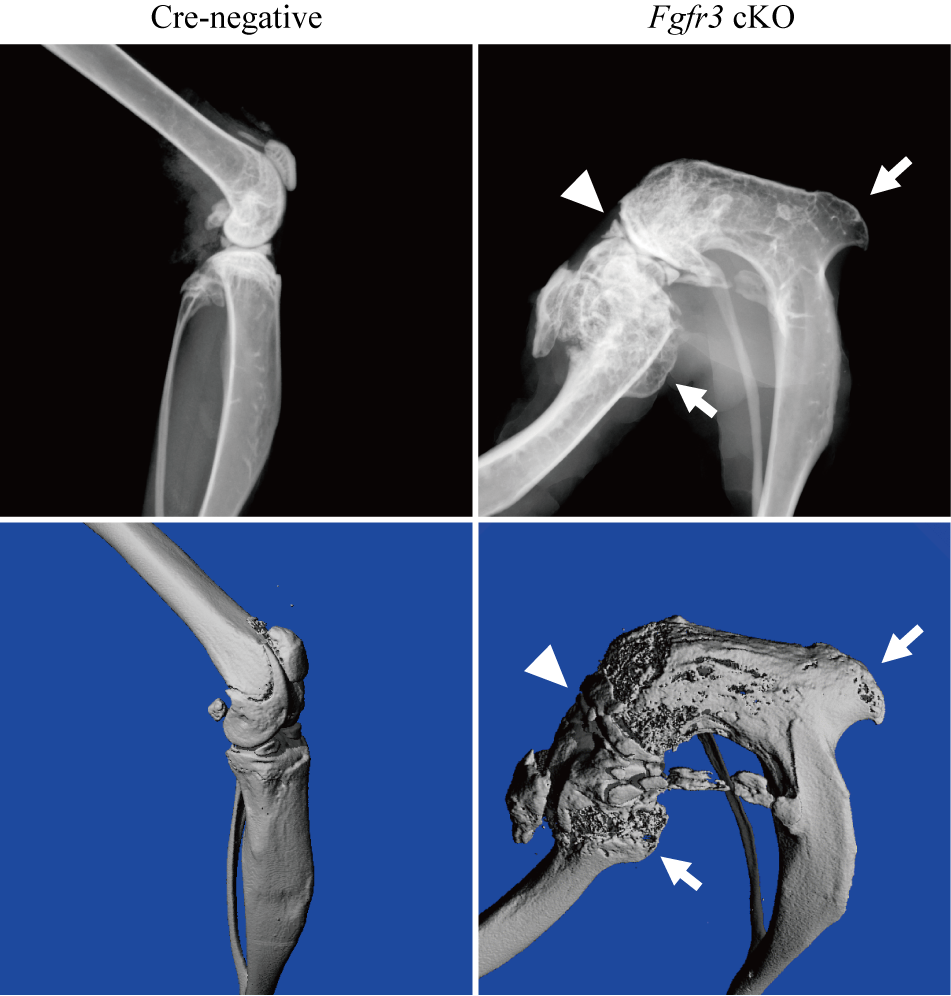

Supplement: S1 Fig — Mice were administered tamoxifen for 8 weeks starting at 4 weeks old and were sacrificed at week 25. X-ray and micro-CT images showing a deformed knee joint (arrowheads) and multiple bony outgrowths (arrows) located near the growth plate in Fgfr3 cKO mice. (TIF) [file pgen.1005214.s001.tif]

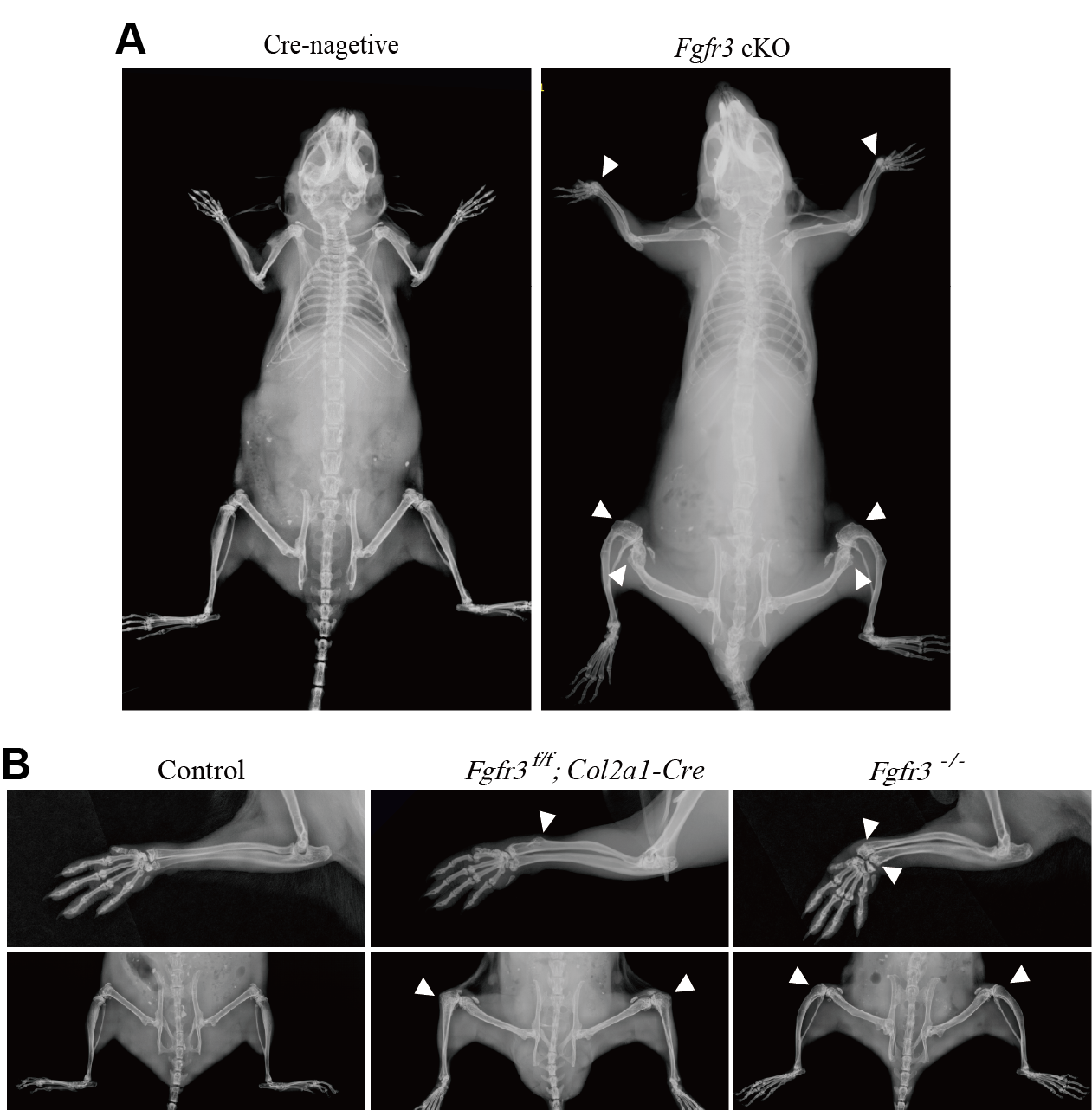

Supplement: S2 Fig — (A) X-ray images of 12-month-old Cre-negative and Fgfr3 cKO mice. Bony lesions were observed around the growth plate of the tibia, femur, ulna, and radius (arrowheads) in mutants. (B) X-ray images of 12-week-old Cre-negative, Fgfr3 f/f; Col2-Cre, and Fgfr3 −/− mice. Multiple bony lesions (arrowheads) were observed in the mutants. (TIF) [file pgen.1005214.s002.tif]

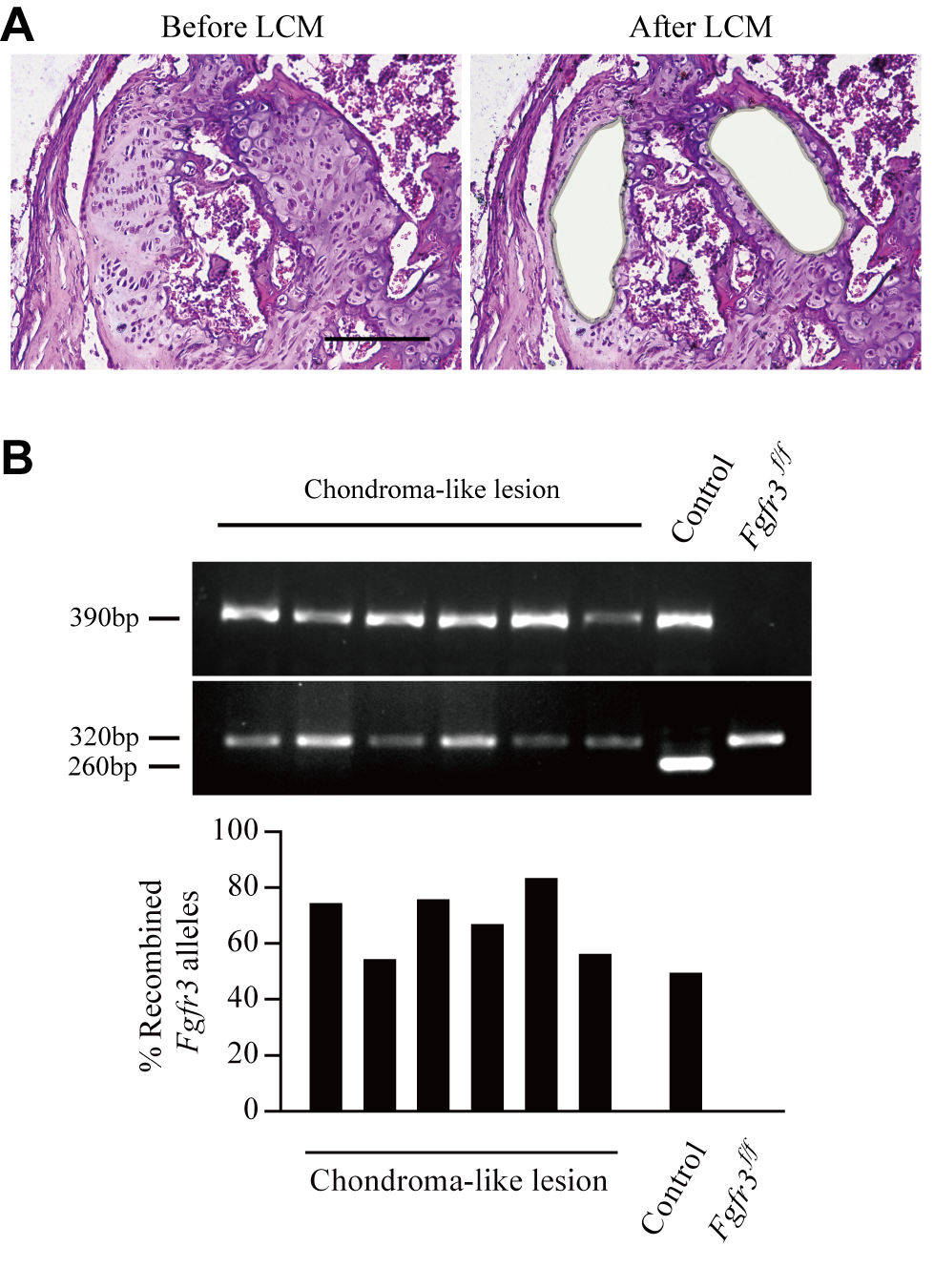

Supplement: S3 Fig — (A) Cartilage tissue of chondroma-like lesions in the tibia isolated by laser-capture microdissection. (B) PCR analysis of DNA from chondroma-like lesions in Fgfr3 cKO mice. DNA from the cartilage of Fgfr3 heterozygous and Cre-negative mice were analyzed as a control. Chondroma-like lesions in Fgfr3 cKO mice contained recombined and unrecombined Fgfr3 alleles; the percentage of recombined Fgfr3 alleles was determined by semiquantitative PCR in chondroma-like lesions of Fgfr3 cKO mice of similar phenotypic severity (n = 6). Recombined Fgfr3 allele = 390 bp; unrecombined Fgfr3 allele = 320 bp; wild-type allele = 260 bp. Scale bar: 200 μm. (TIF) [file pgen.1005214.s003.tif]

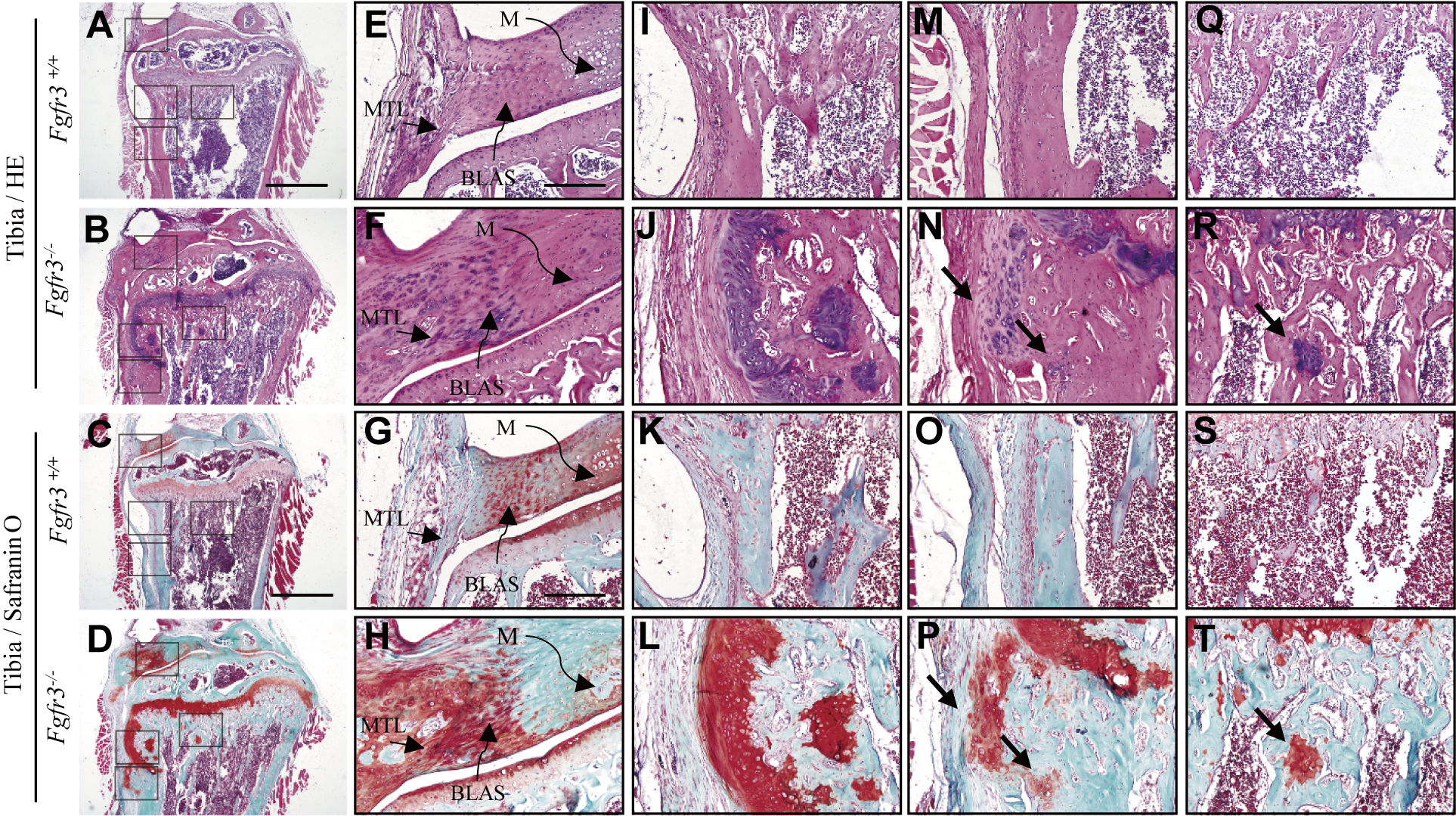

Supplement: S4 Fig — Tissue sections of tibia were stained with Fast Green/Safranin O and by H & E. (A–D) Ectopic cartilage and chondroma-like lesions are observed in Fgfr3 −/− but not in Fgfr3 +/+ mice. Higher magnification views of the areas shown in boxes in E–T. (E–H) Ectopic cartilage at the bone-ligament attachment site (BLAS) of the meniscus (M) and menisco-tibial ligament (MTL) in Fgfr3 −/− mice. (I–P) Osteochondroma-like lesion structurally similar to growth plate cartilage adjacent to ectopic cartilage (arrows) in Fgfr3 −/− mice. (Q–T) Enchondroma-like lesion (arrows) embedded in the trabecular bone of Fgfr3 −/− mice. Scale bar: 1 mm (A–D), 200 μm (E–T). (TIF) [file pgen.1005214.s004.tif]

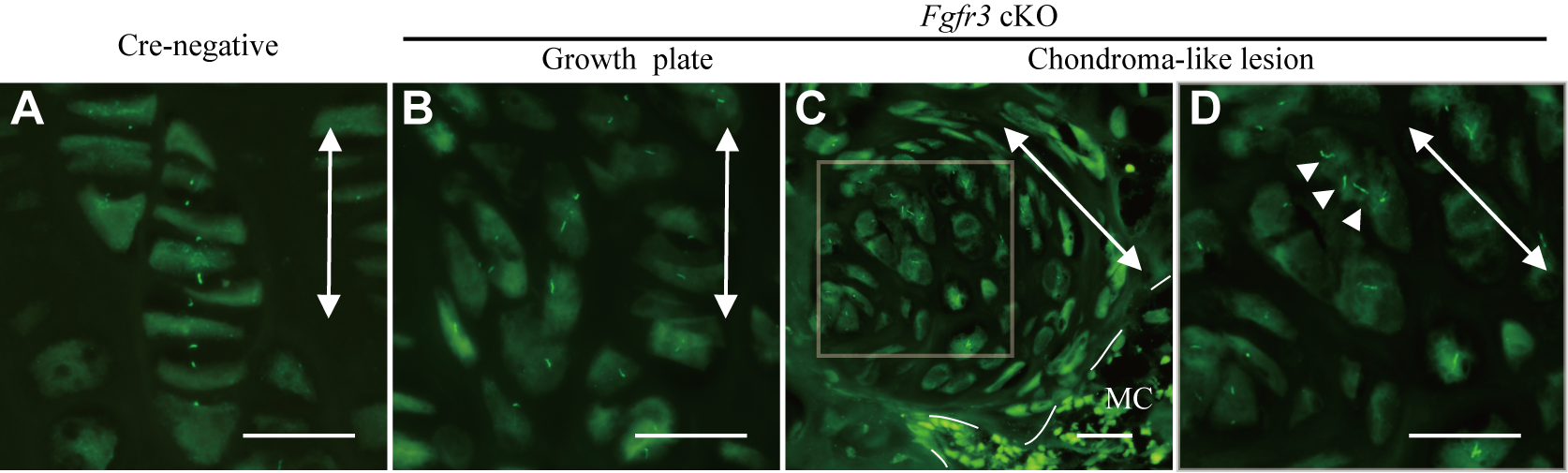

Supplement: S5 Fig — (A) Primary cilia (green line) were observed on the chondrocyte surface extending into the extracellular matrix of cartilage while those in the proliferative zone were parallel to the vertical axis of the growth plate in Cre-negative mice. (B) Primary cilia in the growth plate cartilage of Fgfr3 cKO mice were misoriented. (C, D) Primary cilia of most chondrocytes in chondroma-like lesions were misoriented; some cells were organized into small columns (arrowheads) oriented perpendicularly to the boundary (broken line) between the chondroma-like lesion and marrow cavity (MC). Higher magnification view of the areas shown in boxes in D. Arrow-bar = vertical axis of the growth plate (A, B) or chondroma-like lesion (C, D). Scale bar: 25 μm. (TIF) [file pgen.1005214.s005.tif]

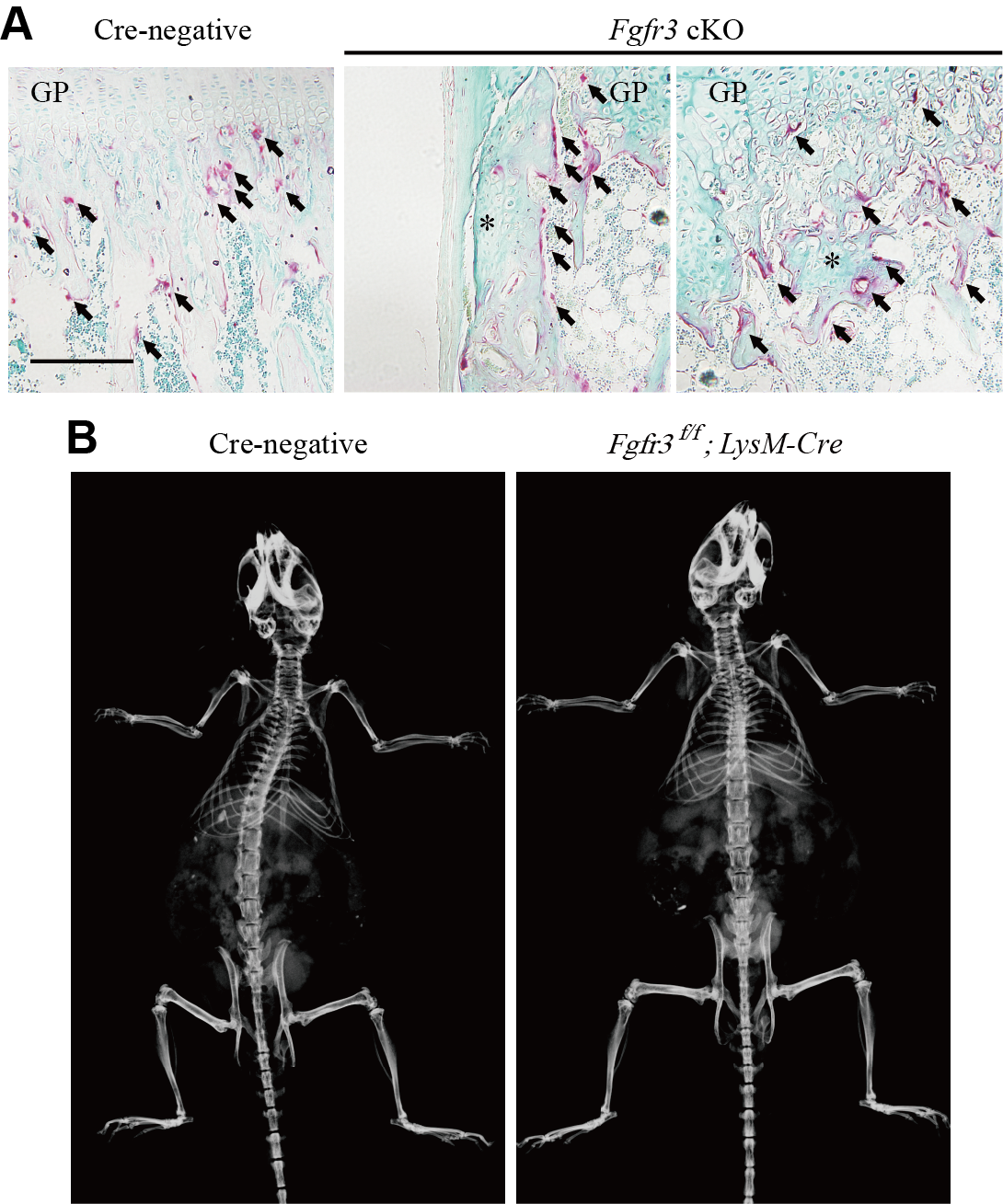

Supplement: S6 Fig — Osteoclast recruitment (arrows) around growth plates (GP) and chondroma-like lesions (asterisks) was unaffected in Fgfr3 cKO mice. (B) X-ray images of 6-month-old Cre-negative and Fgfr3 f/f; lysM-Cre mice. There was no joint deformation or bony lesions observed in Fgfr3 f/f; lysM-Cre mice. Scale bar; 200 μm (A). (TIF) [file pgen.1005214.s006.tif]

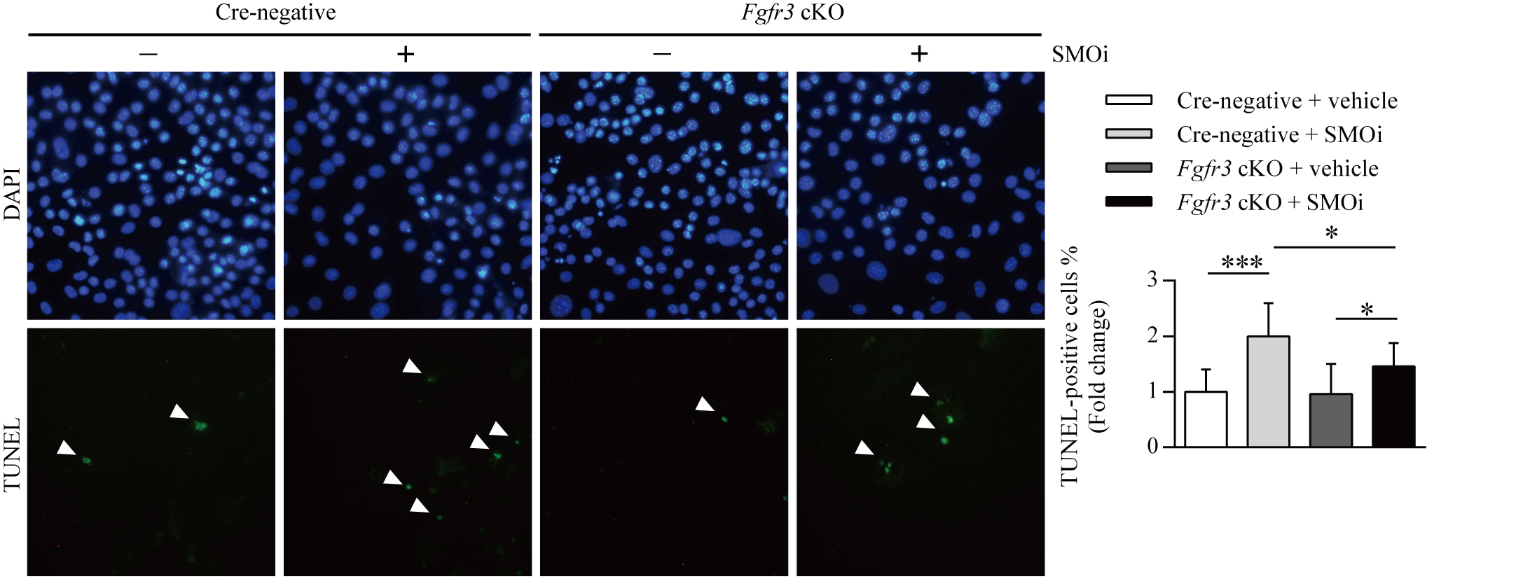

Supplement: S7 Fig — Apoptosis in primary chondrocytes from Cre-negative mice treated with vehicle (dimethyl sulfoxide) or SMOi (GDC-0449) and Fgfr3 cKO mice treated with vehicle or SMOi was detected by TUNEL. Nuclei were visualized by DAPI staining. TUNEL-positive cells (arrows) in primary chondrocytes were counted and are expressed as a percentage of the total number of cells. Values represent mean ± SD. *p < 0.05, ***p < 0.001 (n = 3). (TIF) [file pgen.1005214.s007.tif]

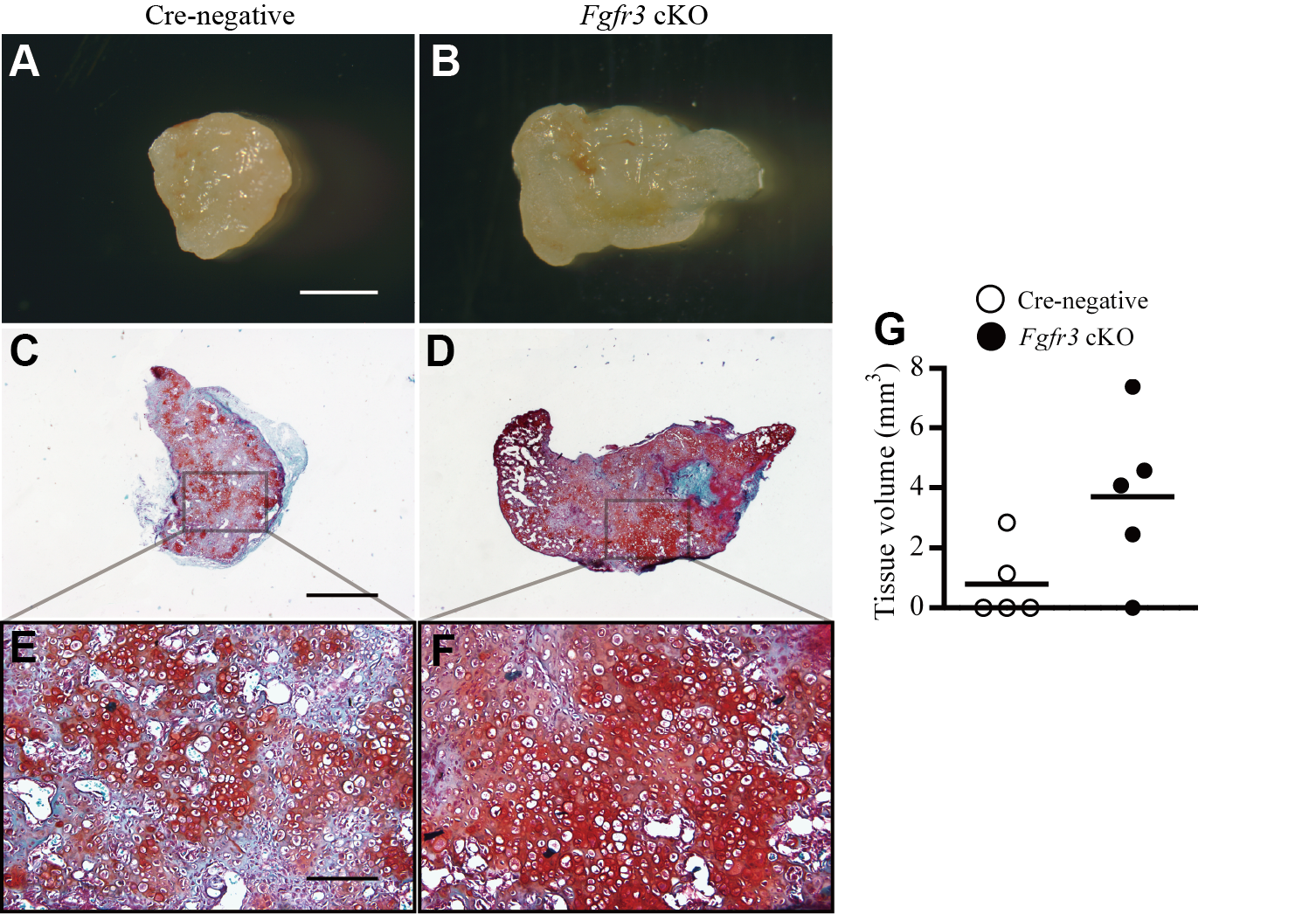

Supplement: S8 Fig — Higher magnification views of areas shown in boxes E and F. Fast Green/Safranin O staining showing a larger area of matrix-enriched chondrocytes in Fgfr3-deficient as compared to Cre-negative chondrocyte transplants. (G) Scatter plots of the volume of recovered chondrocyte transplants. Scale bar: 1 mm (A–D), 200 μm (E, F). (TIF) [file pgen.1005214.s008.tif]

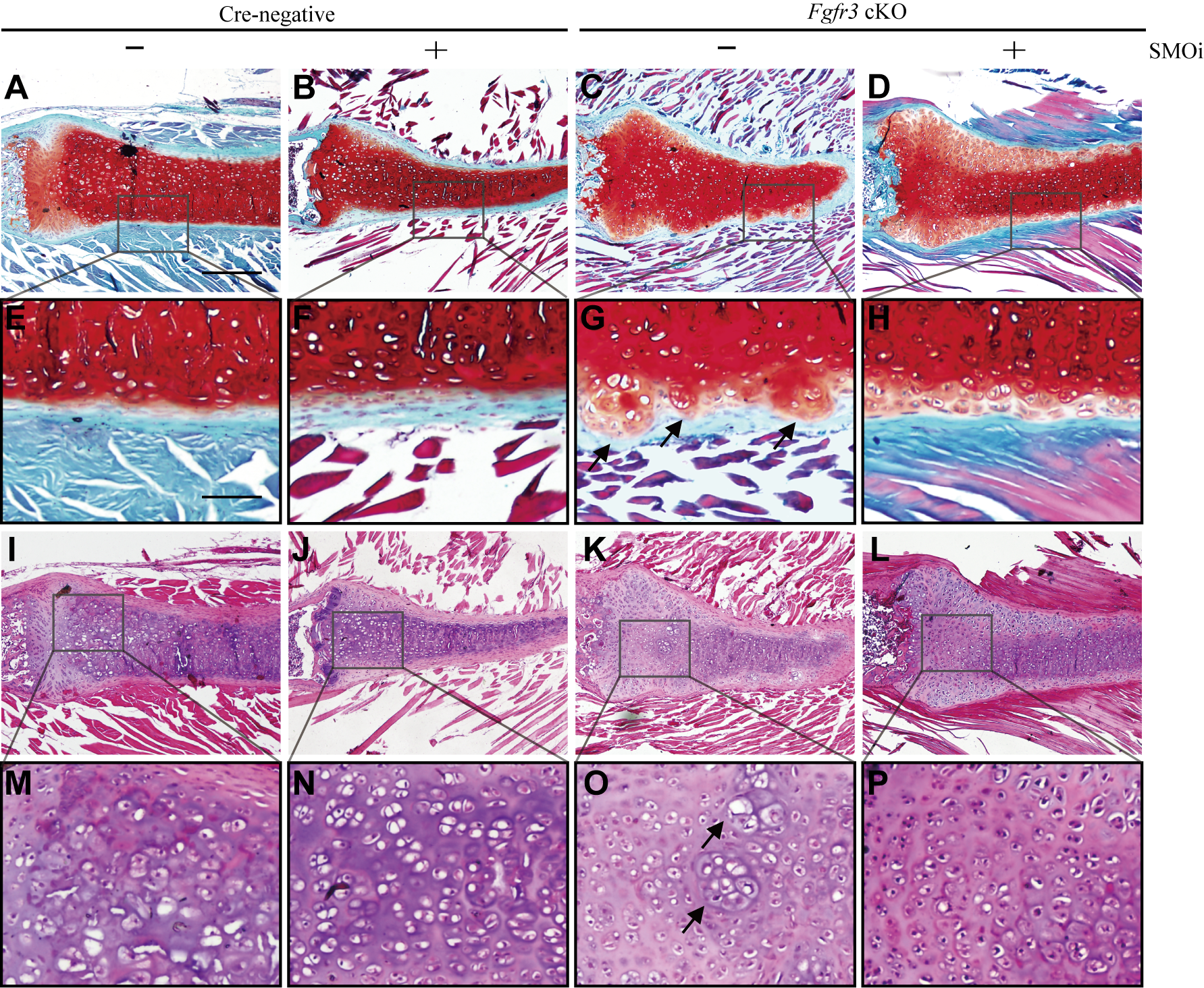

Supplement: S9 Fig — Fast Green/Safranin O staining of the costal cartilage in Cre-negative mice treated with vehicle (n = 9) or SMOi (n = 8) and Fgfr3 cKO mice treated with vehicle (n = 9) or SMOi (n = 8). (A, B, E, F) In Cre-negative mice treated with vehicle or SMOi, the costal cartilage is separated from the surrounding muscle by a line of perichondrium. (C, D, G, H) Hypertrophic chondrocyte clusters (arrows) disrupt perichondrium organization in Fgfr3 cKO mice, this phenotype that was attenuated by SMOi treatment. (I, J, M, N) Cre-negative mice treated with vehicle or SMOi had chondrocytes that were evenly distributed in the middle of the costal cartilage. (K, L, O, P) Formation of hypertrophic chondrocyte clusters (arrows) in the middle of the costal cartilage in Fgfr3 cKO mice was also reduced by SMOi treatment. Scale bar: 400 μm (A–D, I–L), 100 μm (E–H, M–P). (TIF) [file pgen.1005214.s009.tif]
